# iTRAQ-based quantitative proteomic analysis reveals potential factors associated with the enhancement of phenazine-1-carboxamide production in *Pseudomonas chlororaphis* P3

**DOI:** 10.1038/srep27393

**Published:** 2016-06-07

**Authors:** Xue-Jie Jin, Hua-Song Peng, Hong-Bo Hu, Xian-Qing Huang, Wei Wang, Xue-Hong Zhang

**Affiliations:** 1 State Key Laboratory of Microbial Metabolism, School of Life Sciences and Biotechnology, Shanghai Jiao Tong University, Shanghai 200240, China

## Abstract

Phenazine-1-carboxamide (PCN), a phenazine derivative, is strongly antagonistic to fungal phytopathogens. *Pseudomonas chlororaphis* HT66 is a PCN-producing, non-pathogenic biocontrol strain, and we obtained the mutant *P. chlororaphis* P3, which produces 4.7 times more PCN than the wild-type HT66 strain. To reveal the cause of PCN production enhancement in P3 and find potential factors related to PCN biosynthesis, an iTRAQ-based quantitative proteomic analysis was used to study the expression changes between the two strains. Of the 452 differentially expressed proteins, most were functionally mapped into PCN biosynthesis pathway or other related metabolisms. The upregulation of proteins, including PhzA/B, PhzD, PhzF, PhzG, and PhzH, involved in PCN biosynthesis was in agreement with the efficient production of PCN in P3. A number of proteins that function primarily in energy production, amino acid metabolism, and secondary metabolism played important roles in PCN biosynthesis. Notably, proteins involved in the uptake and conversion of phosphate, inorganic nitrogen sources, and iron improved the PCN production. Furthermore, the type VI secretion system may participate in the secretion or/and indirect biosynthetic regulation of PCN in *P. chlororaphis*. This study provides valuable clues to better understand the biosynthesis, excretion and regulation of PCN in *Pseudomonas* and also provides potential gene targets for further engineering high-yield strains.

Natural phenazines, a class of colorful nitrogen-containing secondary metabolites, are widely used to biologically control various fungal phytopathogens mainly because of their broad-spectrum antibiotic properties^1^,^2^,^3^. The biosynthetic mechanisms and high production of phenazines in various microorganisms have been the focus of pesticide research because of their environmental compatibility and the feasibility of genetic engineering^4^,^5^,^6^.

Phenazine-1-carboxamide (PCN) is a phenazine derivative that can be synthesized by several *Pseudomonas* strains, including *Pseudomonas chlororaphis* PCL1391^7^, *Pseudomonas aeruginosa* PAO1^8^, and MML2212^9^, and is a potent biopesticide. For example, PCN produced by *P. chlororaphis* PCL 1391 showed higher antifungal activity against *Fusarium oxysporum* than another phenazine derivative, phenazine-1-carboxylic acid (PCA)^7^. The rice pathogens *Rhizoctonia solani* Kühn and *Xanthomonas oryzae* pv. *oryzae* are inhibited by PCN extracted from *P. aeruginosa* MML2212, and the inhibitory effect is superior to that of carbendazim and rifamycin^9^. Thus, PCN is a promising agricultural antibiotic. Nevertheless, the large-scale production of PCN by *Pseudomonas* strains to achieve commercial objectives has been restricted mainly because of their low yields.

Recently, strategies based on genetic modifications^4^,^5^, have been used to develop strains capable of producing large quantities of phenazines. In addition, genome and comparative transcriptome analyses have revealed potential factors influencing phenazine biosynthesis in *Pseudomonas*^10^,^11^. However, less research has been performed to determine the substantial changes affecting phenazine production at the protein expression level.

Isolated from the rice rhizosphere, *Pseudomonas chlororaphis* HT66 is a non-pathogenic rhizobacterium that exhibits wide-spectrum resistance to fungal phytopathogens because of its capability to produce PCN. However, its PCN production in King’s B (KB) medium was very low, approximately 0.42 g/L after 60 h of growth (Fig. 1b). In our previous work, a high PCN-producing strain, *P. chlororaphis* P3, was obtained by subjecting strain HT66 to multiple rounds of chemical mutagenesis and selection, and total of 138 genes were identified to occur point mutation(s) in the genome of P3 (Supplementary Table S1). The mutant P3 could produce 2.01 g/L of PCN in KB medium after 60 h of growth (Fig. 1b), which was 4.7 times that of HT66. Although HT66 genomic information has been studied through sequencing and a comparative genome analysis^10^, and point mutations in the genome of mutant P3 were identified, the physiological changes that resulted in the increased PCN yield in strain P3 are unclear. Thus, the goal of this work was to reveal the cause of high PCN production in mutant P3 from an expression level and find some potential factors related to PCN biosynthesis. In this study, the isobaric tag for relative and absolute quantification (iTRAQ) technique was applied to study the expression level changes in the proteome between the wild-type strain HT66 and the mutant strain P3. The results of iTRAQ could provide useful clues to understand the biosynthesis, excretion and regulation of PCN in *Pseudomonas* and also provide potential gene targets for further genetic modification to acquire high-yield strains.

## Results

### Overview of the proteomic analysis

**Figure 2:**
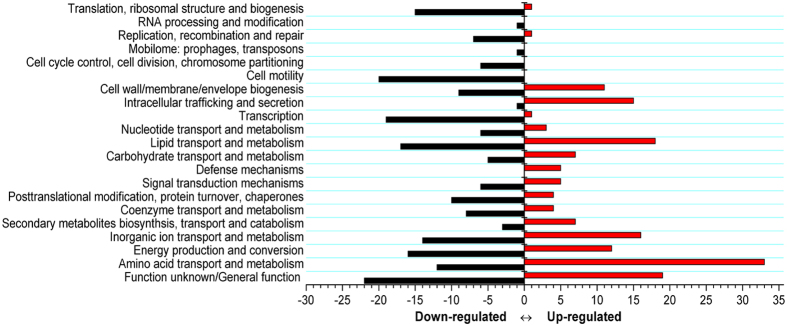
COG classifications of proteins with a fold change of at least 1.5. COG classifications were constructed according to *Pseudomonas chlororaphis* HT66 project in the Integrated Microbial Genomes database (Project ID: Gp0042620). Red and black bars represent the numbers of upregulated and downregulated proteins, respectively.

To understand the physiological changes resulting in an increased PCN yield in mutant P3, the changes in the cellular proteome between the wild-type HT66 and mutant P3 were detected using an iTRAQ analysis. A total of 452 proteins (Supplementary Table S2) were differentially expressed (fold change >1.5 or <0.7, P < 0.05), of which 181 proteins were upregulated and 271 proteins were downregulated. These differentially expressed proteins were profiled based on COG functional classifications. They were categorized into 21 COGs (Fig. 2), indicative of a drastic physiological change, as well as a global response to fluxes in the mutant strain P3. Except for proteins classified into clusters of “Function unknown” or “General function prediction only”, proteins displaying obvious changes in abundance levels were classified into “Amino acid transport and metabolism”, “Energy production and conversion”, “Inorganic ion transport and metabolism”, “Lipid transport and metabolism”, “Intracellular trafficking secretion and vesicular transport”, “Secondary metabolism, transport and catabolism”, and “Transcription”. Most of these categories are associated with higher amounts of PCA in *P. aeruginosa* M18^11^. Thus, such COG clusters were likely to enhance PCN production in P3. Most of the proteins in these COGs were mapped into the PCN biosynthesis pathway or other related metabolisms (as described below). In addition, a large proportion of differentially expressed proteins were assigned to “Cell motility”, “Translation”, “Ribosomal structure and biogenesis”, and “Cell wall/membrane/envelope biogenesis”, indicative of a substantial overall phenotypic difference resulting from the chemical mutagenesis of the mutant P3 compared with the wild-type strain HT66.

### Quantitative RT-PCR and LacZ fusion validation

**Figure 3:**
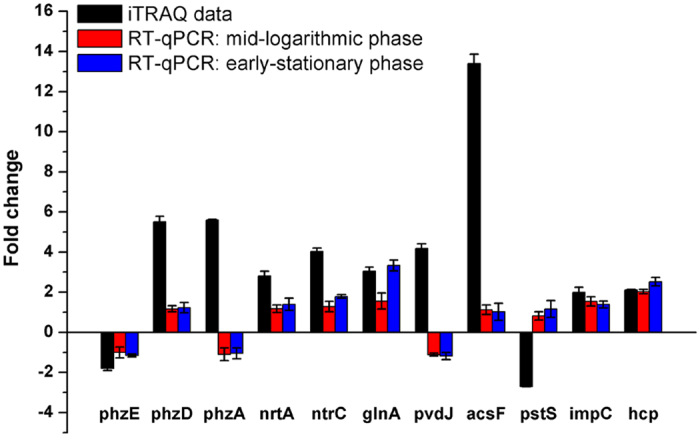
Transcriptional validation of proteomic data using RT-qPCR assays. The value of fold change on the *y*-axis indicates the ratio of either protein (black bar) or mRNA level at the mid-logarithmic phase (red bar) and early-stationary phase (blue bar).

We selected 11 genes, encoding significantly expressed proteins for qRT-PCR validation: *phzA*, *phzE*, and *phzD*, which encode enzymes involved in PCN biosynthesis, *pstS*, which encodes one of the transporters of inorganic phosphate and was downregulated, Pchs_00644 and *pvdJ*, which are related to iron homeostasis and were upregulated, *nrtA*, *ntrC*, and *glnA*, which encode enzymes involved in inorganic nitrogen metabolism and were upregulated, and *impC* and *hcp*, which encode proteins functioned in HS1 and HS2 of type VI secretion system (T6SS) and were upregulated. As shown in Fig. 3, except for *ntrC* and *glnA*, which were upregulated at the early-stationary growth phase, and *impC* and *hcp*, which were upregulated at the mid-logarithmic and early-stationary growth phase, differential expressions of the other genes were not detected.

**Figure 4:**
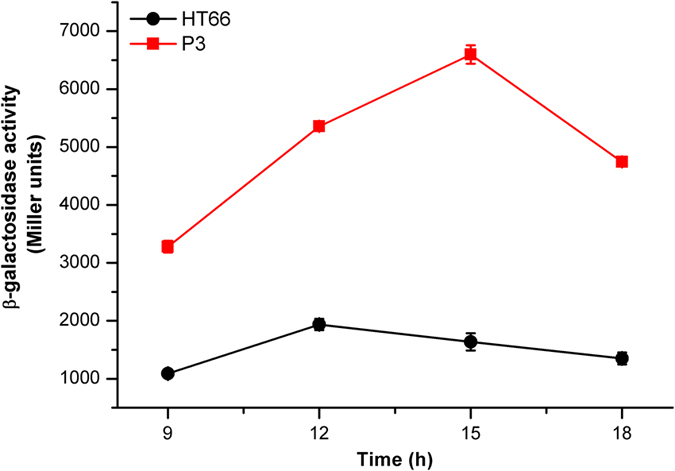
Analysis of β-galactosidase expression from the pBBR-*phz*′-′*lacZ* translational fusion plasmid in the wild-type strain HT66 and the mutant P3 in KB.

To further assess the expression of the *phz* gene cluster in strains HT66 and P3 at the translational level, we constructed a *lacZ* reporter plasmid, pBBR-*phz′-′lacZ* (translational fusion). The β-galactosidase (LacZ) activities resulting from the plasmid were assayed using KB medium for HT66 and P3 strains. We observed that the β-galactosidase activity from the pBBR-*phz′-′lacZ* translational fusion plasmid in mutant P3 was significantly higher (3.01–4.03-fold) than that of strain HT66 (Fig. 4), suggesting that the expression of the *phz* gene cluster was enhanced at the translational level in P3, which was in agreement with the iTRAQ results.

### Specific expression changes related to PCN production

**Figure 5:**
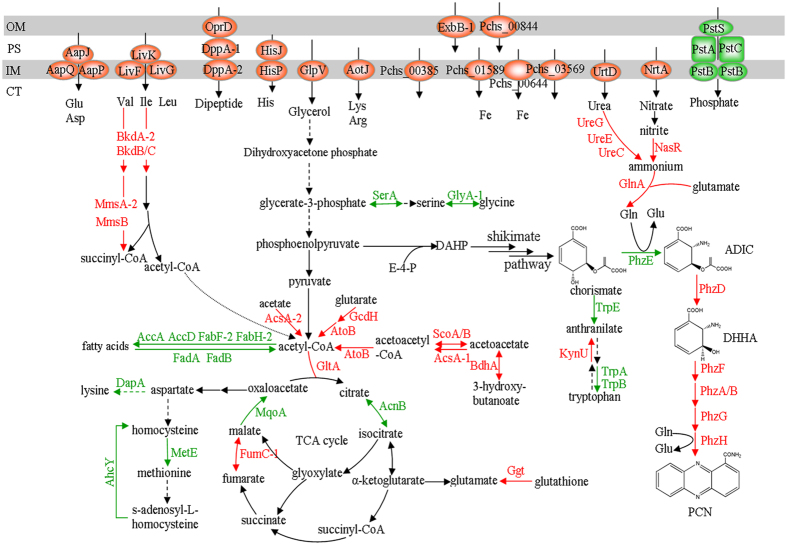
Schematic overview of differentially expressed proteins involved in phenazine-1-carboxamide biosynthesis pathway and related metabolisms, as derived from the iTRAQ analysis. The cell envelope is shown at the top, including relevant porins, proteins, or transporters (OM, outer membrane; PS, periplasmic space; IM, inner membrane; CT, cytoplasm). Some steps are simplified and may be accomplished by several enzymes acting sequentially. Upregulated proteins are marked in red, and downregulated proteins are marked in green. Some proteins without names are indicated by gene locus tags from *P. chlororaphis* HT66. Arrows in black correspond to unaffected steps in P3. The abbreviations used are: E-4-P, erythrose 4-phosphate; DAHP, 3-deoxy-D-*arabino*- heptulosonate 7-phosphate; ADIC, 2-amino-4-deoxychorismic acid; DHHA, *trans*-2,3-dihydro-3-hydroxyanthranilic acid; PCN, phenazine-1-carboxamide; TCA, tricarboxylic acids.

According to the iTRAQ analysis, a number of differentially expressed proteins in mutant P3 enhanced PCN production. These proteins were mapped into PCN biosynthesis pathway or other related metabolisms, including primary metabolism (amino acid metabolism and energy metabolism), secondary metabolism, uptake and conversion of inorganic nutrients (phosphate, iron, and inorganic nitrogen sources), and secretion systems (Table 1; Fig. 5).

### Changes in PCN biosynthesis

In the phenazine-1-carboximide biosynthetic operon, *phzABCDEFGH*, the expression change of PhzC protein was not detected. With the exception of PhzE protein, which was downregulated slightly (0.56-fold), the other proteins involved in PCN biosynthesis, including PhzA/B (5.6-/4.48-fold, respectively), PhzD (5.5-fold), PhzF (1.7-fold), PhzG (2.58-fold), and PhzH (3.33-fold) were significantly upregulated (Table 1; Fig. 5). In order to detect the importance of PhzE in the PCN biosynthesis, we overexpressed the *phzE* gene in mutant P3, resulting in 18–21% increase of the PCN yield in P3 (Supplementary Figure S1), which suggests that PhzE is crucial for PCN biosynthesis. Overall, the above results are in good agreement with the efficient production of PCN in P3.

### Changes in energy metabolism

In *P. chlororaphis* P3, many proteins involved in energy metabolism were differentially expressed (Table 1; Fig. 5). GlpV, a putative transporter responsible for the transport of glycerol, was upregulated significantly (8.16-fold). Four proteins (GltA, AcnB, FumC-1, and MqoA) in the citric acid (TCA) cycle were expressed differentially. The upregulation of proteins, AcsA-1/-2, GcdH, AtoB, BdhA, and ScoA/B, involved in the degradation of acetate, glutarate, and 3-hydroxybutanoate was observed in P3. Certain proteins involved in the biosynthesis and oxidation of fatty acids, AccA, AccD, FabH-2, FabF-2, and FadA/B, were downregulated.

### Changes in amino acid metabolism

Numerous proteins involved in amino acid metabolism and transport were expressed differentially in mutant P3 (Table 1; Fig. 5). Proteins involved in the transport of lysine and arginine (AotJ), valine, leucine, and isoleucine (LivF/G and LivK), histidine (HisP and HisJ), and dipeptides (OprD and DppA-1/-2) were upregulated. Other proteins, identified as a putative amino acid ABC transporter (Pchs_00385) and different components of less well-characterized amino acid transporters (AapP/Q and AapJ), were also upregulated. Changes in some amino acid transporters resulted in the changes in corresponding metabolisms, such as the downregulation of lysine biosynthesis (DapA) and the upregulation of valine and isoleucine (BkdA-2, BkdB/C, MmsA-2, and MmsB) assimilation. Two proteins (MetE and AhcY) and one transcriptional regulator (MetR) of methionine biosynthesis were downregulated, which was consistent with the oxaloacetate decrease in the TCA cycle, resulting from the downregulation of protein MqoA. The serine and glycine biosynthesis pathway was downregulated because of the decreased expression of proteins involved in this pathway (SerA and Gly-1). In addition, the expression of protein Ggt, responsible for converting glutathione to glutamate, and GlnA, responsible for converting glutamate to glutamine, were upregulated.

In addition to the proteins involved in general amino acid metabolisms, three proteins (TrpA/B and TrpE) involved in the tryptophan biosynthesis pathway, a main competing pathway of PCN biosynthesis, were downregulated (Fig. 5). One of the proteins involved in tryptophan catabolism was upregulated (KynU).

### Changes in secondary metabolism

Some proteins involved in secondary metabolism were differentially expressed. As shown in Table 1, OfaB, one of the proteins involved in the biosynthesis of the secondary metabolite orfamide, was downregulated. The expression levels of proteins, including two chitinases (ChiC, Pchs_02788 and Pchs_03281) and two chitin-binding proteins (CbpD, Pchs_02789 and Pchs_03280), contributing to fungal resistance in *Pseudomonas* were significantly downregulated (Table 1). Such results probably favor PCN biosynthesis because the competition for carbon fluxes was reduced. Furthermore, some proteins involved in the biosynthesis of secondary siderophores, pyoverdine and achromobactin, were significantly upregulated (Table 1), which may improve PCN production. The specific explanation for this was reported in the “Changes in iron uptake” section.

### Changes in the uptake and conversion of inorganic nutrients

#### Changes in phosphate uptake

**Figure 6:**
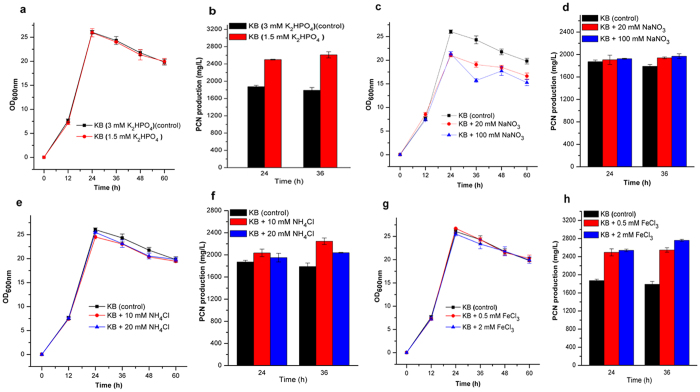
Influence of inorganic ions on the growth and phenazine-1-carboxamide (PCN) production. The growth and PCN production of mutant P3 in KB medium amended with different concentrations of K_2_HPO_4_ (a**,b**), NaNO_3_ (**c,d**), NH_4_Cl (**e,f**); and FeCl_3_ (**g,h**), respectively. Experiments were performed at least three times with similar results. Data of one of the experiments are shown.

Phosphate, an essential nutrient for the growth of most microorganisms, is an important environment factor that affects PCN production in *P. chlororaphis* PCL1391^12^. In the iTRAQ proteomic results, we observed the significant downregulation of the transporter PstSCAB-PhoU, which is involved in phosphate uptake and the downregulation of PhoB, a central regulator that is activated under phosphate-limited conditions (Table 1 and Fig. 5). It is hypothesized that reducing the phosphate concentration in KB would increase the PCN level. Thus, the KB media amended with 1.5 mM K_2_HPO_4_ was used to monitor the growth and PCN production of the wild-type strain HT66 and mutant P3, with KB media (containing 3 mM K_2_HPO_4_) as the control. Consistent with the iTRAQ findings, reducing K_2_HPO_4_ concentration enhanced the production of PCN in P3 and HT66 (Fig. 6b and Supplementary Figure S2b). PCN production of P3 was 2.61 g/L after 36 h of culture with KB media amended with 1.5 mM K_2_HPO_4_, resulting in a 45.8% increase in PCN level compared with 1.79 g/L PCN of the control (KB with 3 mM K_2_HPO_4_).

#### Changes in nitrate uptake and conversion

As shown in Table 1 and Fig. 5, the assimilation of nitrogen increased because of the upregulation of some proteins involved in this pathway, including NrtA, a nitrate transporter, protein NasR, responsible for conversion of nitrite to ammonia, and glutamine synthetase (GlnA), which catalyzes the reaction of ammonia with glutamate to form glutamine. Two regulatory proteins, GlnK and NtrC, were also upregulated. These proteins were essential in activating the expression of genes participating in glutamine biosynthesis in the *Pseudomonas* strains^13^,^14^. In addition, UrtD, a urea transporter, and three proteins (UreC, UreE, and UreG) identified to be urease or urease accessory proteins, responsible for converting urea to ammonium, were upregulated. Thus, we speculated that the addition of nitrate or ammonia to the medium would stimulate PCN production in P3. To test this assumption, HT66 and P3 cells were grown in KB media supplemented with different concentrations of NaNO_3_ (20 and 100 mM, respectively) or NH_4_Cl (10 and 20 mM, respectively) to determine PCN production. The growth and production of PCN in P3 are shown in Fig. 6c–f. The addition of 20 or 100 mM NaNO_3_ to the KB media resulted in a reduction of the P3′ OD_600_ value at the stationary phase (Fig. 6c), while the PCN production showed a 8.4% or 10% increase, respectively, at 36 h of incubation (Fig. 6d). The addition of 100 mM NaNO_3_ to the KB media stimulated the production of PCN in HT66 at 36 h of incubation (Supplementary Figure S2d). The addition of 10 or 20 mM NH_4_Cl resulted in a 25.5% or 13.9% increase in the PCN level in P3 at 36 h of incubation (Fig. 6e,f) while PCN level decreased in HT66 (Supplementary Figure S2f).

#### Changes in iron uptake

**Figure 7:**
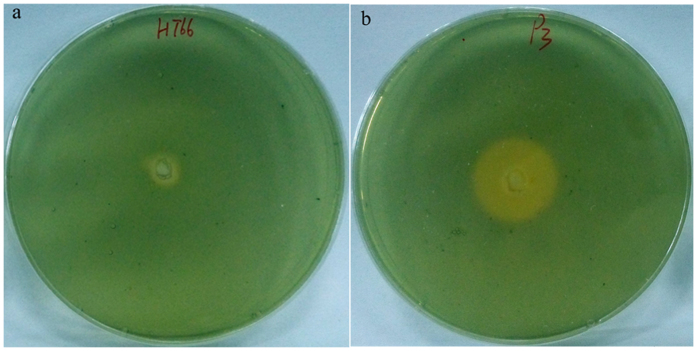
Siderophore biosynthesis assay. Siderophore production in the wild-type strain HT66 (**a**) and in the mutant P3 (**b**) was detected on CAS blue agar plates. The strains were incubated for 24 h at 28 °C. The level of siderophore production is indicated by the size of the orange zone.

Proteins involved in iron homeostasis were significantly upregulated in P3 (Table 1 and Fig. 5). Among these proteins, five proteins involved in pyoverdine biosynthesis and six proteins involved in achromobactin biosynthesis were upregulated. Pyoverdine and achromobactin are secondary siderophores mainly responsible for iron uptake from the extracellular environment for cell growth and metabolism in *Pseudomonas*^15^,^16^. In addition, many proteins identified as TonB-dependent receptors were also upregulated. TonB-dependent receptors have been characterized in fluorescent pseudomonads and are required for the uptake of siderophores^17^. Two proteins, ExbB-1 and Pchs_01590, involved in iron uptake were upregulated as well. Another protein, Fur, was downregulated slightly. This protein is a transcriptional repressor of the expression of genes involved in the synthesis of siderophores in pseudomonads^18^. Based on these results, we hypothesized that P3 could produce more siderophores than HT66 and that iron is vital for improving PCN production. Therefore, we first measured the siderophores produced by strain HT66 and mutant P3 using CAS solid plates. The diameter of the fluorescent region around the inoculation point, which reflects siderophores production, was significantly wider in P3 than in HT66 after growth at 28 °C for 24 h (Fig. 7a,b).

Furthermore, to investigate the influence of iron on the PCN production of strain HT66 and mutant P3, the growth and PCN production in two strains in KB media supplemented with 0.5 or 2.0 mM FeCl_3_ were monitored. KB media (without iron) was the control. As shown in Fig. 6h, when P3 was cultured in the KB media amended with 0.5 or 2.0 mM FeCl_3_, the PCN production was 2.55 and 2.76 g/L at 36 h of incubation, resulting in a 42.3% or 54.2% increase in the PCN level, respectively. Conversely, the addition of 0.5 or 2.0 mM FeCl_3_ to the KB media resulted in a decrease in the PCN level in HT66 (Supplementary Figure S2h). It is indicated that change of proteins involved in iron homeostasis may be one of factors resulting in the enhancement of PCN production in mutant P3.

#### Changes in secretion systems

The iTRAQ results showed that multiple proteins related to secretion were differentially expressed. Five proteins identified as components of the type I secretion system (T1SS) were upregulated (Table 1). In most Gram-negative bacteria, T1SS is responsible for translocating various proteins with known and unknown functions from the cytoplasm to the extracellular milieu^19^. In addition to the T1SS proteins, 10 proteins identified as subunits of the T6SS were also upregulated in P3 (Table 1). Gene clusters encoding T6SS components have been found in various *Pseudomonas* species^20^. Some *Pseudomonas* strains encode more than one T6SS, for example, three T6SSs, H1-T6SS to H3-T6SS, have been described in *P. aeruginosa*^21^. Previous studies have reported that some secreted T6SS effectors play crucial roles in *Pseudomonas* virulence and competition, and various T6SSs within a single strain may serve different functions^22^,^23^. However, the function of T6SS in *P. chlororaphis* is still not clear. The HT66 genome sequence alignment showed that HT66 contains three distinct homologous T6SS loci, identified as HS1, HS2, and HS3. Among the 10 proteins expressed differentially, one (Pchs_01292) was independent in the genome, two (Pchs_04376 and Pchs_04382) were subunits of HS1, and the other seven were subunits of HS2. The change in protein expression of HS3 was not detected while a point mutation in the gene encoding the IcmF protein, which is a subunit of HS3, was found (Supplementary Table S1). Therefore, we speculated that T6SS may participate in the secretion and/or indirect biosynthetic regulation of PCN in our strains. The three gene clusters, HS1-HS3, encoding T6SS machinery were deleted in HT66 and P3 to assess the PCN production.

**Figure 8:**
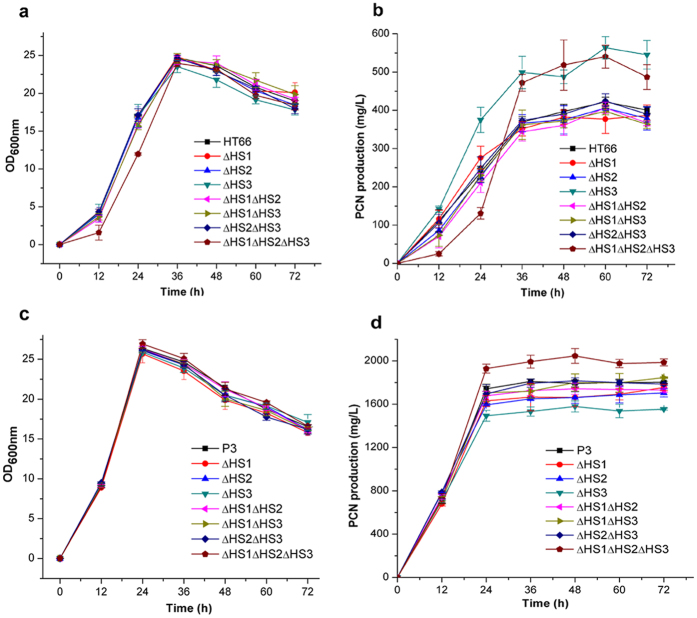
The growth and phenazine-1-carboxamide (PCN) production of mutants deleted T6SS. The growth (**a,c**), and PCN production (**b,d**) of mutants based on the wild-type strain HT66 and mutant P3, respectively. Experiments were performed at least three times with similar results. Data of one of the experiments are shown.

To better understand the roles of each locus, we generated large fragment deletions of HS1, HS2, and HS3 by removing 20.7, 24.6, and 20.3 kb DNA segments. As shown in Fig. 8, simultaneous deletion of three gene clusters or deletion of only HS3 gene locus in HT66 resulted in an obvious increase in the PCN level. Similar to HT66, simultaneous deletion of three gene clusters in P3 resulted in an obvious increase in the PCN level. Conversely, deletion of only HS3 gene locus in P3 resulted in an obvious decrease in the PCN level. Such difference may derive from the change of genomes of these two strains. Also, deletion of only HS1 or HS2 in HT66 and P3 resulted in different degrees of decrease in the PCN level. The above results indicated that T6SS may participate in the secretion and/or biosynthetic regulation of PCN in our strains and each locus of the T6SS probably plays an independent role.

## Disscusion

In this paper, we investigated the proteome of a high PCN-producing mutant P3 and made an analysis of the proteomic differences between the wild-type strain HT66 and mutant P3 to reveal the cause of high PCN production in P3. The differential expression of 452 proteins suggested a drastic physiological change as well as a global response to the change of fluxes in the P3 mutant. The significant expression changes of proteins involved in biosynthesis of PCN were line with the efficient production in P3. Many differentially expressed proteins contribute to PCN production, including proteins involved in primary metabolism (amino acid and energy metabolism), secondary metabolism and uptake and conversion of inorganic ions (phosphate, iron and inorganic nitrogen). Of note, T6SS probably plays an important role in the secretion of PCN or regulation of PCN biosynthesis in *P. chlororaphis*.

The PCN biosynthetic operon, *phzABCDEFGH*, has been well characterized in *P. chlororaphis* PCL1391^24^ and *P. aeruginosa* PAO1^8^. Mavrodi *et al.*^25^ found that five genes, encoding the proteins PhzA, PhzD, PhzE, PhzF, and PhzG, with the *phzA* gene duplicated as *phzB* in pseudomonads, are essential for the biosynthesis of the phenazine scaffold in all phenazine-producing pseudomonads. As enzymes catalyzing the biosynthesis of PCN, the upregulation of proteins PhzA/B, PhzD, PhzF, PhzG and PhzH was line with the efficient PCN production in P3. PhzE is a anthranilate synthase that catalyzes the first step in the biosynthesis of phenazine and is responsible for converting chorismate, the end product of the shikimate pathway, and ammonia supplied by L-glutamine, into 2-amino-4-deoxychorismic acid (ADIC)^2^,^26^,^27^. It is crucial for enhancement of PCN production of mutant P3.

In KB media, glycerol is the main carbon source, and peptone including various amino acids and peptides is the main nitrogen source^28^. The upregulation of GlpV suggests that more glycerol is absorbed and enters the central carbon metabolism^29^. This facilitates cell growth as well as favors the enhancement of PCN production because part of carbon flux derived from glycerol enters phenazine biosynthesis pathway (Fig. 5). The upregulation of proteins responsible for transport and utilization of isoleucine and valine indicates the increase of the levels of the TCA cycle intermediate succinyl-CoA and acetyl-CoA that feeds directly into the TCA cycle. Differential expression of proteins involved in energy metabolism indicates that the acetyl-CoA level increased and more carbon fluxed towards the TCA cycle. These results suggest that mutant P3 could produce sufficient energy to fuel its growth and proliferation, which is helpful for PCN biosynthesis as well.

In the present study, we found that the biosynthesis pathway of serine and glycine weakened. This pathway competes for metabolic flux towards the shikimate pathway (Fig. 5). Thus, this result contributes to increase the metabolic flux into shikimate pathway, and improves PCN production because the phenazine precursors are derived from the shikimate pathway^1^,^26^. As a competing pathway, the expression downregulation of tryptophan pathway suggests that more chorismate, the common precursor of biosynthesis of PCN and tryptophan, tends to be used to synthesize PCN (Fig. 5). Jin *et al.*^5^ found that PCA production of *P. aeruginosa* PA1201 was improved by increasing the metabolic flux towards the shikimate pathway, and blocking the tryptophan pathway by deleting the *trpE* gene resulted in a 15% increase of the PCA level in PA1201.

The production of antibiotic phenazines is profoundly regulated or stimulated by many environmental factors and mineral nutrients^12^,^30^,^31^. Notably, our iTRAQ results showed that multiple proteins involved in uptake and/or utilization of phosphate, iron and inorganic nitrogen ions were differentially expressed in mutant P3. The downregulation of proteins involved in the transport of phosphate implies that lower phosphate favors the enhancement of PCN production. Similar to *P. chlororaphis* PCL1391^12^, in our strains, lower concentration PO_4_^3−^ resulted in an increase of PCN level. The upregulation of proteins involved in nitrogen metabolism (NrtA, NasR, GlnK, NtrC and GlnA) and urea metabolism (UrtD, UreC, UreE, and UreG) implies that intracellular glutamine level increased, which likely contributes to improvement of PCN level because glutamine provides an amino group for chorismate to form ADIC in phenazines biosynthesis^26^,^32^,^33^. van Rij, E. T. *et al.* reported that different nitrogen sources, including NaNO_3_, NH_4_Cl and glutamine, greatly influenced PCN levels in *P. chlororaphis* PCL1391^14^. The fermentation results showed that NaNO_3_ had stimulatory effect on PCN production in our strains. The upregulation of proteins involved in iron homeostasis likely contributes to PCN biosynthesis because we found that addition of FeCl_3_ in medium greatly improved PCN production in mutant P3. Previous reports showed that ferric iron (Fe^3+^) has a positive effect on the production of PCA in *P. chlororaphis* PCL1391^12^ and *P. fluorescens* 2-79^30^. Further research is needed to elucidate the specific mechanisms.

As relatively stable antibiotics, most of phenazines produced by *Pseudomonas* are secreted into the culture medium during fermentation^25^. Therefore, the secretion system plays an important role in promoting phenazines production. According to previous studies, intracellular PCA levels activate *mexGHI-opmD* gene in *P. aeruginosa* M18^11^, and overexpression of this gene in *P. aeruginosa* PA1201 significantly increased PCA production, indicating that the products of *mexGHI-opmD* gene function as a PCA efflux pump^5^. In our iTRAQ results, the change of proteins such as efflux pumps encoded by *mexGHI-opmD*, was not detected, while multiple proteins related to secretion were differentially expressed in T6SS. The deletion of gene clusters of T6SS in our strains indicates each locus of the T6SS probably plays an independent role. Further studies are required to investigate the complex relevance of HS1, HS2, and HS3 in the secretion and/or biosynthetic regulation of PCN.

In conclusion, we identified multiple proteins related to enhancement of PCN production by iTRAQ-base quantitative proteomic analysis. These identified proteins may provide useful clues to better understand the biosynthesis, regulation, and excretion of PCN in *Pseudomonas* and also provide potential gene targets for genetic modification to acquire high-yield strains.

## Methods

### Strains and culture media

All strains and plasmids used in this study are listed in Table 2. Luria–Bertani (LB) medium (tryptone 10.0 g, yeast extract 5.0 g, NaCl 10.0 g/L) was used for the incubation of *E. coli* and *P. chlororaphis* during the construction of mutants. *E. coli* was incubated at 37 °C, while *P. chlororaphis* was cultured at 28 °C. KB medium (glycerol 20 g, tryptone 20 g, MgSO_4_ 0.732 g, K_2_HPO_4_ 0.514 g/L) was used for activating *P. chlororaphis* HT66 and P3 during iTRAQ and fermentation experiments.

### Bacteria cultivation and protein preparation

*P. chlororaphis* HT66 and P3 cells were inoculated into 60 mL of KB liquid medium in a 250-mL flask at 28 °C with shaking at 180 rpm. When the bacteria reached the early stationary growth phase, culture samples were collected by centrifuging at 10000 g for 10 min at 4 °C to extract total proteins using the protocol described by Liu *et al.*^34^. The proteins were quantified using the BCA Protein Assay Kit (Thermo Scientific, Rockford, IL, USA).

### Protein digestion and iTRAQ labeling

For each sample, 200 μg of protein was reduced by incubation with 1 M dithiothreitol (20 mM final concentration) at 56 °C for 1 h. Cysteine residues were blocked using 1 M indole-3-acetic acid (90 mM final concentration) for 40 min at room temperature in the dark. Proteins were digested overnight by Sequencing Grade Modified Trypsin (Promega, Madison, WI, USA) in 50 mM NH_4_HCO_3_ in a 1:50 trypsin-to-protein mass-ratio.

For each digested sample, 100 μg was labeled at room temperature for 3 h with 4-plex iTRAQ reagents (AB Sciex, Framingham, MA, USA). iTRAQ reagents 114 and 116 were used to label two peptide samples of HT66 as the controls, and iTRAQ reagents 115 and 117 were used to label two peptide samples from P3. The four labeled peptides were combined in a 1:1:1:1 ratio (v/v) and dried by Speed Vac.

### HPLC peptide fractionation and Nano LC-MS/MS analysis

The peptide mixture was re-dissolved in buffer A (20 mM ammonium formate, pH 10.0), and then fractionated using a Survey HPLC system (Thermo Scientific, Waltham, MA, USA) by a reverse-phase column (Agela, Durashell-C18 Column, 2.1 mm × 250 mm, 5 μm, 100 Å). A total of 39 fractions of approximately 1 mL were collected and then combined into 12 fractions based on peak intensities. The protein identification was performed using an UltiMate 3000 Nano-LC system (Dionex, Thermo Fisher Scientific, MA, USA) coupled with an ESI-Q-TOF mass spectrometer (maXis, Impact, Bruker Daltonik, Germany).

### Database search and data analysis

The MS/MS data were used as query by Mascot 2.4.1 (Matrix Science, London, UK) against the P_ht66_20140926 database (unknown version, 6,362 entries), assuming the digestion enzyme was trypsin. The criteria for the database search included a fragment ion mass tolerance of 0.05 Da and a mass tolerance of 20 ppm. Peptide charges of +2, +3, and +4 were selected. The carbamidomethyl of cysteine, iTRAQ 4-plex of lysine, and the N-terminus were specified in Mascot as fixed modifications. The oxidation of methionine and iTRAQ 4-plex of tyrosine were specified in Mascot as variable modifications.

The isobaric tagged peptides and proteins were identified with Scaffold Q+ (version 4.3.4; Proteome Software Inc., Portland, OR, USA). Peptide identifications were accepted if they had a probability greater than 94.0% to achieve a false discovery rate (FDR) of less than 1.0% using the Scaffold Local FDR algorithm. Protein identifications were accepted if they had a greater than a 98.0% probability to achieve a FDR of less than 5.0% and contained at least two identified peptides^35^. Proteins with a 1.5-fold change when comparing mutant P3 against the control HT66, and a p-value of statistical evaluation less than 0.05 were considered as differentially expressed proteins. The functional classification of identified proteins was conducted using the Integrated Microbial Genomes database (https://img.jgi.doe.gov/cgi-bin/er/main.cgi) against *P. chlororaphis* HT66. The biological pathways were established using the KEGG Pathway Database (http://www.genome.jp/kegg/) and the literature.

### Quantitative real-time PCR

Culture samples at the mid-logarithmic and early-stationary growth phases were collected for RT-qPCR analysis. Briefly, total RNAs were extracted from cells of HT66 and P3 using a TRIzol total RNA isolation reagent (Invitrogen, Carlsbad, CA, USA), and reverse transcribed to cDNA using SuperScript II reverse transcriptase (Invitrogen). RT-qPCR was then carried out on an ABI ViiA 7 real-time PCR system (Applied Biosystems, Foster City, CA, USA) using the miScript SYBR Green PCR Kit (Qiagen, Shanghai, China). The gene-specific primers were designed using ABI primer express software v2.0 (Supplementary Table S1) and the *rpoD* gene encoding sigma70 was used as a reference^36^. The fold change for mRNAs was calculated by the 2^−ΔΔCt^ method^37^.

### Construction of the *lacZ* fusion plasmid and overexpression plasmid of *phzE*

To assess the differential expressions of the *phz* gene cluster in HT66 and P3, pBBR1MCS^38^ was used in the construction of the *lacZ* translational fusion. The rrnBT1 terminator amplified from plasmid pBbB5k-GFP^39^ was first fused with a 524-bp fragment containing the promoter/operator region and the first several codons of the *phz* gene cluster, generating a T1*phz* fragment. A *LacZ* fragment deleting promoter and first eight codons of the *E. coli lacZ* gene was amplified from plasmid pME6015^40^. Then, the T1*phz* fragment and *lacZ* fragment were simultaneously inserted into the plasmid pBBR1MCS using an In-Fusion HD Cloning Kit (Takara Bio Inc., Shiga, Japan) to generate a pBBR-*phz′-′lacZ* translational fusion plasmid. The plasmid was transformed into *P. chlororaphis* by electroporation.

The fragment of *phzE* was amplified from genome of P3 and inserted into the plasmid pBBR1MCS using an In-Fusion HD Cloning Kit (Takara Bio Inc., Shiga, Japan), generating a pBBR-phzE plasmid. pBBR-phzE and pBBR1MCS (as control) were transformed into P3, respectively, by electroporation to determine PCN production. Expression was induced with 0.5 mM IPTG.

### Quantification of β-galactosidase activity

Overnight cultures of strains HT66 and P3 harboring the *lacZ* reporter plasmid were inoculated into 250-mL Erlenmeyer flasks containing 60 mL KB supplemented with kanamycin sulfate to an initial optical density at 600 nm (OD_600_) of 0.02. Cultures were incubated at 28 °C with shaking at 200 rpm for 24 h. At different stages of growth, the cells were harvested to assay β-galactosidase activities using the method of Miller^41^.

### *In vitro* assay for siderophore production

The *P. chlororaphis* siderophore production *in vitro* assay was conducted on chrome azurol S (CAS) agar solid medium as described by Schwyn and Neilands^42^. A hole, 0.5 cm in diameter, was excavated from the center of the CAS plate. An inoculum (10 μL) of strain HT66 or P3 was dropped into the hole, and the plate was cultured at 28 °C for 24 h. Siderophore production was assessed by the formation of a distinct fluorescent orange zone on the CAS plate.

### Quantitative assay for PCN production

Fermentation broth (400 μL) was acidified to pH 2.0 with 6 M HCl, and then 3.6 mL of ethyl acetate was added. The sample was vigorously agitated and centrifuged at 13,000 g for 5 min. A 400-μL portion of the upper layer was collected and evaporated in a rotary evaporator. The residue was dissolved in 1 mL acetonitrile. PCN concentrations were determined by HPLC (Agilent Technologies 1200 series, Santa Clara, USA) using a calibration curve. HPLC was performed using a C18 reverse-phase column (Agilent Eclipse, XDB-C18, 4.6 mm × 250 mm, 5 μm, Santa Clara, USA) with an UV light detector using a solution of 8% acetonitrile and 92% 5 mM NH_4_Ac as the mobile phase at a flow rate of 1 mL/min.

### Inorganic ions influence the PCN production

The effects of differentially expressed proteins involved in the uptake and conversion of phosphate, iron, and nitrogen sources, on PCN production were assessed by growing strain P3 in KB medium supplemented with different concentrations of corresponding ions. The influence of phosphate on PCN production was determined by the addition of 1.5 mM K_2_HPO_4_ to KB medium. KB medium, containing 3 mM K_2_HPO_4_, was used as the control. The influence of iron was measured by the addition of 0.5 mM or 2.0 mM FeCl_3_ to the KB medium. To determine the effects of nitrogen sources on PCN production, two strains were cultured in KB medium supplemented with NaNO_3_ (20 mM or 100 mM) or NH_4_Cl (10 mM or 20 mM). PCN production was measured by HPLC as described above. Cell growth was determined by measuring the absorbance at 600 nm (OD_600_).

### Non-scar deletion of three gene clusters of T6SS

Three gene clusters encoding proteins of T6SS were deleted using the non-scar deletion method described by Du *et al.*^4^. Briefly, two DNA fragments flanking HS1, HS2, and HS3, were amplified by PCR from the genome, and then, they were combined using overlap PCR. The non-scar modified DNA fragment was cloned into pK18mobsacB, and the resulting plasmid was then transferred into *E. coli* S17-1 (λpir) to be mobilized into HT66 and P3 by biparental mating. The clones occurring as a single-crossover event were selected from plates containing 100 μg/mL ampicillin and 50 μg/mL kanamycin, and then clones occurring as a double-crossover events were selected from plates containing 15% sucrose and 50 μg/mL kanamycin. The clones were confirmed by PCR analysis and sequencing. All the primers used to delete gene clusters are shown in Table S3.

## Additional Information

**How to cite this article**: Jin, X.-J. *et al.* iTRAQ-based quantitative proteomic analysis reveals potential factors associated with the enhancement of phenazine-1-carboxamide production in *Pseudomonas chlororaphis* P3. *Sci. Rep.* **6**, 27393; doi: 10.1038/srep27393 (2016).

## Supplementary Material

Supplementary Information

## Figures and Tables

**Figure 1:**
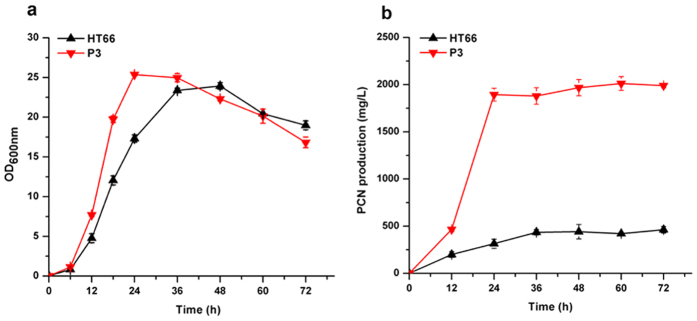
The growth (**a**) and phenazine-1-carboxamide production (**b**) of *P. chlororaphis* HT66 and mutant strain P3.

**Table 1:** Differentially expressed proteins associated with PCN production in *P. chlororaphis* P3.

Protein description	Gene	IMG locus tag_HT66	NCBI GI_HT66	Ratio^a^
Phenazine-1-carboxamide biosynthesis				
Phenazine biosynthesis protein A/B	*phzA*	Pchs_06091	647490047	5.60
Phenazine biosynthesis protein A/B	*phzB*	Pchs_06092	647490048	4.48
Isochorismate hydrolase	*phzD*	Pchs_06094	647490052	5.52
Anthranilate/para-aminobenzoate synthases component I	*phzE*	Pchs_06095	647490054	0.56
Phenazine biosynthesis protein PhzF family	*phzF*	Pchs_06096	647490057	1.78
Pyridoxamine 5′-phosphate oxidase	*phzG*	Pchs_06097	647490058	2.58
Asparagine synthase	*phzH*	Pchs_06098	647490062	3.33
Aromatic amino acid metabolism				
Tryptophan synthase, alpha chain	*trpA*	Pchs_00084	647491730	0.54
Tryptophan synthase, beta chain	*trpB*	Pchs_00083	496337064	0.30
Anthranilate synthase, component I	*trpE*	Pchs_00718	647493343	0.67
Kynureninase	*kynU*	Pchs_01436	647499417	1.88
General amino acid metabolism				
Phosphoglycerate dehydrogenase and related dehydrogenases	*serA*	Pchs_00455	495208070	0.43
Serine hydroxymethyltransferase	*glyA_1*	Pchs_06270	496341782	0.53
Dihydrodipicolinate synthase/N-acetylneuraminate lyase	*dapA*	Pchs_02172	647483598	0.40
Methionine synthase (B12-independent)	*metE*	Pchs_05826	647489417	0.34
Transcriptional regulator, LysR family	*metR*	Pchs_05827	496341406	0.6
Pyruvate/2-oxoglutarate dehydrogenase complex, dehydrogenase (E1) component	*bkdA_2*	Pchs_04155	647498492	2.27
Branched-chain alpha-keto acid dehydrogenase E2 component	*bkdB*	Pchs_04156	647498496	2.21
Dihydrolipoamide dehydrogenase	*bkdC*	Pchs_04157	647498498	2.10
Methylmalonate-semialdehyde dehydrogenase	*mmsA_2*	Pchs_01422	647499383	2.34
3-hydroxyisobutyrate dehydrogenase	*mmsB*	Pchs_01423	647499387	1.70
TCA cycles				
Citrate synthase	*gltA*	Pchs_02520	496338587	3.05
Aconitase	*acnB*	Pchs_04041	647498241	0.6
Fumarase, class II	*fumC_1*	Pchs_01637	647490465	5.57
Malate:quinone-oxidoreductase	*mqoA*	Pchs_04769	647485851	0.52
Fatty acid metabolism				
Acetyl-CoA carboxylase carboxyltransferase subunit alpha	*accA*	Pchs_01907	495199263	0.57
Beta-ketoacyl-acyl-carrier-protein synthase II	*fabF_2*	Pchs_02742	647484125	0.41
3-oxoacyl-[acyl-carrier-protein] synthase III	*fabH_2*	Pchs_02430	647487188	0.53
3-ketoacyl-CoA thiolase	*fadA*	Pchs_02879	496338784	0.59
Short chain enoyl-CoA hydratase	*fadB*	Pchs_02880	647484600	0.62
Acetyl-CoA carboxylase carboxyltransferase subunit alpha	*accD*	Pchs_02808	647484424	0.67
Acetate, glutarate, and 3-hydroxybutanoate catabolism				
Acetoacetyl-CoA synthase	*acsA_1*	Pchs_01546	647499663	2.42
Acyl-CoA dehydrogenases	*gcdH*	Pchs_00003	647491551	2.19
3-hydroxybutyrate dehydrogenase	*bdhA*	Pchs_01547	647499665	4.5
Acetyl-coenzyme A synthetase	*acsA_2*	Pchs_06191	647487629	2.32
3-oxoacid CoA-transferase, A subunit ScoA	*scoA*	Pchs_03129	495195793	71.6
3-oxoacid CoA-transferase, B subunit ScoB	*scoB*	Pchs_03130	495195791	7.49
Acetyl-CoA acetyltransferase	*atoB*	Pchs_03131	647487719	9.64
Secondary metabolism				
Long-chain-fatty-acid–CoA ligase	*ofaB*	Pchs_04408	647500074	0.36
Chitinase	*chiC*	Pchs_02788	647484393	0.12
Chitin-binding protein	*cbpD*	Pchs_02789	647484396	0.08
Chitin-binding protein	*cbpD*	Pchs_03280	647496507	0.15
Chitinase	*chiC*	Pchs_03281	647496510	0.2
Achromobactin biosynthesis				
TonB-dependent ferric achromobactin receptor protein	–	Pchs_03383	647496719	4.5
Achromobactin biosynthesis protein AcsF	*acsF*	Pchs_03384	647496722	13.41
Achromobactin biosynthesis protein AcsD	*acsD*	Pchs_03385	647496724	4.28
Achromobactin biosynthesis protein AcsA	*acsA*	Pchs_03390	647496739	7.35
Siderophore achromobactin ABC transporter, ATPase component	*fepC*	Pchs_03394	647496750	2.85
Putative achromobactin biosynthesis protein	–	Pchs_03396	647496754	2.32
Pyoverdine biosynthesis				
Acyl-homoserine lactone acylase PvdQ (EC 3.5.1.-), quorum-quenching	*pvdQ*	Pchs_03685	647497454	3.06
Putative dipeptidase, pyoverdin biosynthesis PvdM	*pvdM*	Pchs_05058	647486584	2.96
PvdO, pyoverdine responsive serine/threonine kinase	*pvdO*	Pchs_05060	647486586	2.55
Pyoverdine sidechain non-ribosomal peptide synthetase PvdD	*pvdD*	Pchs_05064	647486599	1.8
Pyoverdine sidechain non-ribosomal peptide synthetase PvdJ	*pvdJ*	Pchs_05153	647493566	4.18
TonB-dependent siderophore receptors				
TonB-dependent siderophore receptor	*fhuA*	Pchs_00644	647493125	15.86
TonB-dependent siderophore receptor	*piuA*	Pchs_01589	647490308	8.56
TonB-dependent siderophore receptor	*fecA*	Pchs_01712	647490614	1.67
TonB-dependent siderophore receptor	–	Pchs_02718	647484077	1.65
TonB-dependent siderophore receptor	–	Pchs_03569	647497170	3.11
TonB-dependent siderophore receptor	–	Pchs_04973	647486375	1.69
TonB-dependent heme/hemoglobin receptor family protein	*phuR*	Pchs_05644	647488959	1.56
Outer membrane transport energization protein ExbB (TC 2.C.1.1.1)	*exbB1*	Pchs_00288	647492252	1.95
Ferric uptake regulation protein FUR	*fur*	Pchs_01489	495196050	0.66
Transport systems				
Carbohydrate ABC transporter substrate-binding protein, CUT1 family (TC 3.A.1.1.-)	*glpV*	Pchs_04820	647485965	8.16
Amino acid ABC transporter substrate-binding protein, PAAT family (TC 3.A.1.3.-)	*aapJ*	Pchs_01762	647490729	7.84
Amino acid ABC transporter membrane protein 1, PAAT family (TC 3.A.1.3.-)	*aapQ*	Pchs_01763	647490732	2.49
Amino acid ABC transporter ATP-binding protein, PAAT family (TC 3.A.1.3.-)	*aapP*	Pchs_01765	496337929	4.7
Amino acid/amide ABC transporter ATP-binding protein 2, HAAT family (TC 3.A.1.4.-)	*livF*	Pchs_00883	496337539	3.21
Amino acid/amide ABC transporter ATP-binding protein 1, HAAT family (TC 3.A.1.4.-)	*livG*	Pchs_00884	565889081	3.35
Amino acid/amide ABC transporter substrate-binding protein, HAAT family (TC 3.A.1.4.-)	*livJ*	Pchs_00887	496337535	3.51
Lysine-arginine-ornithine-binding periplasmic protein	*aotJ*	Pchs_04910	647486203	3.73
Amino acid ABC transporter substrate-binding protein, PAAT family (TC 3.A.1.3.-)	–	Pchs_00385	647492551	5.60
ABC-type dipeptide transport system, periplasmic component	*dppA_1*	Pchs_01603	647490337	1.78
Outer membrane porin, OprD family	*oprD*	Pchs_01604	647490339	3.1
ABC-type dipeptide transport system, periplasmic component	*dppA_2*	Pchs_01605	647490341	2.02
Amino acid ABC transporter ATP-binding protein, PAAT family (TC 3.A.1.3.-)	*hisP*	Pchs_02011	647491334	1.58
Amino acid ABC transporter substrate-binding protein, PAAT family (TC 3.A.1.3.-)	*hisJ*	Pchs_02015	496338179	1.65
Phosphate transport				
Phosphate ABC transporter substrate-binding protein, PhoT family (TC 3.A.1.7.1)	*pstS*	Pchs_00239	495203739	0.37
Phosphate transport system permease protein PstC (TC 3.A.1.7.1)	*pstC*	Pchs_00240	647492140	0.54
Phosphate transport system permease protein PstA (TC 3.A.1.7.1)	*pstA*	Pchs_00241	496342427	0.58
Phosphate transport ATP-binding protein PstB (TC 3.A.1.7.1)	*pstB*	Pchs_00242	496342426	0.54
Phosphate transport system regulatory protein PhoU	*phoU*	Pchs_00243	495203747	0.65
Phosphate regulon transcriptional regulatory protein PhoB	*phoB*	Pchs_00249	495171678	0.65
Uncharacterized protein conserved in bacteria	–	Pchs_00250	647492159	0.59
Nitrate uptake and metabolism (Nitrogen metabolism)				
Nitrate ABC transporter, nitrate-binding protein	*nrtA*	Pchs_02944	647484724	2.81
Assimilatory nitrite reductase (NAD(P)H) large subunit precursor (EC 1.7.1.4)	*nasR*	Pchs_02949	647484728	3.23
L-glutamine synthetase (EC 6.3.1.2)	*glnA*	Pchs_01089	647495467	3.05
Nitrogen regulatory protein P-II family	*glnK*	Pchs_00342	488618959	2.25
Nitrogen regulation protein NR(I)	*ntrC*	Pchs_01102	647495504	4.04
Urea ABC transporter, ATP-binding protein UrtD	*urtD*	Pchs_00853	647494818	2.48
Urease subunit alpha [EC:3.5.1.5]	*ureC*	Pchs_00860	647494842	1.83
Urease accessory protein UreE	*ureE*	Pchs_00876	647494888	2.64
Urease accessory protein UreG	*ureG*	Pchs_00878	496337543	2.79
Secretion systems				
Type I secretion C-terminal target domain (VC_A0849 subclass)	*lapA*	Pchs_01388	647496163	1.71
Type I secretion membrane fusion protein, HlyD family	*hlyD*	Pchs_02366	647487032	2.19
Type I secretion system ATPase, LssB family	*lapB*	Pchs_02367	647487034	2.33
Type I secretion outer membrane protein, TolC family	*tolC*	Pchs_02368	647487037	2.90
VCBS repeat/type I secretion C-terminal target domain (VC_A0849 subclass)	*lapA*	Pchs_02369	647487039	2.66
Type VI secretion protein, VC_A0107 family	*impB*	Pchs_00257	495205505	2.09
Type VI secretion protein, EvpB/VC_A0108 family	*impC*	Pchs_00258	495205504	2.22
Type VI secretion protein, VC_A0110 family	*impG*	Pchs_00265	647492201	2.50
Type VI secretion ATPase, ClpV1 family	*clpV*	Pchs_00267	647492206	1.77
Type VI secretion protein, VC_A0114 family	*impJ*	Pchs_00272	495205486	1.74
Type IV/VI secretion system protein, DotU family	*dotU*	Pchs_00273	647492220	1.65
Type VI secretion protein IcmF	*icmF*	Pchs_00274	647492224	1.99
Type VI secretion system effector, Hcp1 family	*hcp*	Pchs_04376	496335162	2.11
Type VI secretion protein, VC_A0114 family	*impJ*	Pchs_04382	647500000	1.53
Type VI secretion system effector, Hcp1 family	*hcp_1*	Pchs_01292	495200970	1.59

^a^Fold change between two strains (mutant P3/control HT66), ratio >1.5 represented upregulation, ratio <0.7 represented downregulation.

**Table 2:** Strains and plasmids used in this study.

Strain/plasmid	Characteristics^a^	Source
Strains		
HT66	*P. chlororaphis* Wild-type, PCN, Ap^r^ Sp^r^	This study
P3	A mutant from HT66 with a high PCN production, Ap^r^ Sp^r^	This study
S17-1(λpir)	*E. coli res- pro mod+ integrated copy of RP4, mob+, used for incorporating constructs into P. chlororaphis*	Lab stock
Plasmids		
pBBR1MCS	T7 expression vector, Km^r^	38
pBbB5k-GFP	LacUV5 expression vector, Km^r^	39
pME6015	pVS1-p15A *E. coli*–*Pseudomonas* shuttle vector for constructing the translational *lacZ* fusions, Tc^r^	40
pBBR-phz’-’lacZ	pBBR1MCS containing a rrnBT1 terminator from pBbB5k-GFP, a 524bp fragment covering from promoter/operator region and the first seven codons of *phz* gene cluster and *lacZ* gene from pME6015, Km^r^	This study
pK18mobsacB	Broad-host-range gene replacement vector; sacB, Km^r^	Lab stock

^a^Ap^r^, Sp^r^, Km^r^ and Tc^r^, ampicillin, spectinomycin, kanamycin and tetracycline resistance, respectively; r, resistance.
