# Supplementary material for: iTRAQ-based quantitative proteomic analysis reveals potential factors associated with the enhancement of phenazine-1-carboxamide production in Pseudomonas chlororaphis P3
Source: Sci Rep. 2016 Jun 7;6:27393. doi: 10.1038/srep27393 (PMC4895345; doi:10.1038/srep27393)
Supplement: Supplementary Information [file srep27393-s1.pdf]

Supplementary Data for:

iTRAQ-based quantitative proteomic analysis reveals potential factors associated with the enhancement of phenazine-1-carboxamide production in *Pseudomonas chlororaphis* P3

Xue-Jie Jin, Hua-Song Peng, Hong-Bo Hu, Xian-Qing Huang, Wei Wang, Xue-Hong Zhang*

State Key Laboratory of Microbial Metabolism, School of Life Sciences and Biotechnology, Shanghai Jiao Tong University, Shanghai 200240, China

*Corresponding author: Tel.: +86-21-3420-6715; fax: +86-21-3420-5081

E-mail: xuehzhang@sjtu.edu.cn

Contents:

Supplementary Tables S1-S3

Supplementary Figures S1-S2

Supplementary Tables

Supplementary Table S1. Total of 138 genes occurred point mutation (s) in the genome of mutant P3. TC represents termination codon.

IMG locus tag_HT66	Gene	Gene product name	nucleotides change	Amino acid change
Pchs_01406		Predicted xylanase/chitin deacetylase	C780T	-
Pchs_06129		putative spermidine/putrescine transport system substrate-binding protein	G98A	R33H
Pchs_05107	*pvdJ*	Non-ribosomal peptide synthetase modules and related proteins	C988T	Q330TC
Pchs_02623		transcriptional regulator, TetR family	G403A	A135T
Pchs_02626	*lysA*	diaminopimelate decarboxylase	G228A	-
Pchs_02632	*rtcA*	RNA 3'-terminal phosphate cyclase (ATP)	C776T	A259V
Pchs_02640		hypothetical protein	C318T, C321T, T438C	-
Pchs_02652		carboxynorspermidine dehydrogenase	C1103T	A368V
Pchs_02660		Predicted flavoprotein CzcO associated with the cation diffusion facilitator CzcD	C71T	A24V
Pchs_02661		transcriptional regulator, AraC family	G735A	-
Pchs_02670		hypothetical protein	G981A	-
Pchs_02674		Purine nucleoside permease	G604A	E202K
Pchs_02682		glycolate oxidase FAD binding subunit	G641A	R214H
Pchs_02730		Acyl-CoA dehydrogenase	G1242A	-
Pchs_02753		amino acid adenylation domain-containing protein	C5098T	P1700S
Pchs_02887	*pdxB*	4-phosphoerythronate dehydrogenase (EC 1.1.1.290)	C489T	D220N
Pchs_02928	*zipA*	cell division protein ZipA	C598T	P200S
Pchs_02108		hypothetical protein	C492T	-
Pchs_02115		Choline/Carnitine o-acyltransferase	G710 to A710	S237N
Pchs_02119		Fatty acid desaturase	G335A	G113R
Pchs_02125		Predicted arabinose efflux permease, MFS family	C1053T	D70N
Pchs_02127		hypothetical protein	C352T	P118S
Pchs_02133		type VI secretion system secreted protein VgrG	G690A, T716C	L239P
Pchs_03137		transcriptional regulator, GntR family	A723G	-
Pchs_04575	*cbtA*	cobalt transporter subunit CbtA	C404T	A135V
Pchs_04589	*yejF*	microcin C transport system ATP-binding	G880A	G235D

		protein		
Pchs_04590	*yejE*	microcin C transport system permease protein	G41A	R14H
Pchs_04595		Outer membrane receptor proteins, mostly Fe transport	G30A, G2854A	A952T
Pchs_04600		Protein N-acetyltransferase, RimJ/RimL family	G123A	P20S
Pchs_04929	*codA*	cytosine deaminase	C131T	P44L
Pchs_05033		uncharacterized protein	G340A	E114K
Pchs_02134		hypothetical protein	C327T	-
Pchs_06595		hypothetical protein	G447A, G483T, A486 C, G498A	E162D, H166Q
Pchs_02221		Integrase	G723A	W241TC
Pchs_02223		Superfamily I DNA or RNA helicase	T497C	V166A
Pchs_02278		MFS transporter, CP family, cyanate transporter	G271A	A91T
Pchs_02305		biotin-dependent carboxylase uncharacterized domain-containing protein	C835T	P279S
Pchs_02308		Uncharacterized protein involved in outer membrane biogenesis	G618A	-
Pchs_02310		beta-hydroxylase	C472T	-
Pchs_02313		outer membrane autotransporter barrel domain-containing protein	G2541A	-
Pchs_02369		type I secretion C-terminal target domain (VC_A0849 subclass)	A3238G	D1080G
Pchs_02395		condensin subunit ScpA	G615A	-
Pchs_02431		Uncharacterized protein conserved in bacteria	C879T	-
Pchs_02435	*fliD*	flagellar hook-associated protein 2	C165T	-
Pchs_02438	*fleQ*	sigma-54 specific transcriptional regulator, flagellar regulatory protein A	C1160T	P387L
Pchs_02439	*fleS*	PAS/PAC sensor signal transduction histidine kinase	C732T	-
Pchs_02441	*fliE*	flagellar hook-basal body complex protein FliE	G78A	-
Pchs_03109		transcriptional regulator, XRE family with cupin sensor	T171C	-
Pchs_03120		sarcosine oxidase, monomeric form	A309G	-
Pchs_03121		Predicted signal-transduction protein containing cAMP-binding and CBS domains	A115G	I39V
Pchs_03130	*scoB*	3-oxoacid CoA-transferase subunit B	A308G	D103G

Pchs_05182	*ectB*	diaminobutyrate aminotransferase apoenzyme (EC 2.6.1.76)	C107T	S36L
Pchs_05189		Protein of unknown function (DUF3077)	C32T	P11L
Pchs_05193	*pvdL*	non-ribosomal peptide synthase domain TIGR01720/amino acid adenylation domain-containing protein	C8534T	A2845V
Pchs_05226		EAL domain, c-di-GMP-specific phosphodiesterase class I (or its enzymatically inactive variant)	G527A	G176D
Pchs_05228	*shlB*	hemolysin activation/secretion protein	G989A	G330E
Pchs_05229	*shlA*	hemolysin	G4149A	-
Pchs_05241	*tpmT*	thiopurine S-methyltransferase	C344T	A115V
Pchs_05243	*deaD*	ATP-dependent RNA helicase DeaD	C593T	A198V
Pchs_05248		Uncharacterized conserved protein YjiS, DUF1127 family	C56T	S19F
Pchs_05285	*kdpE*	two component transcriptional regulator, winged helix family	C532T	Q178TC
Pchs_05294		transcriptional regulator, GntR family	C92T	A31V
Pchs_05296		hypothetical protein	C508T	L170F
Pchs_05311	*algA*	mannose-1-phosphate guanylyltransferase / mannose-6-phosphate isomerase	G1057A	V353I
Pchs_05333		ATP-binding cassette, subfamily C	G1192A	A398T
Pchs_05342	*mnmC*	tRNA 5-methylaminomethyl-2-thiouridine biosynthesis bifunctional protein	G525T	-
Pchs_05345		GNAT-family acetyltransferase TIGR03103	C1598T	A533V
Pchs_05347		hypothetical protein	C166T	P56S
Pchs_05350		hypothetical protein Pfl01_4107	C13T	-
Pchs_05355	*atuH*	citronellyl-CoA synthetase	G879A	W293TC
Pchs_05356		Protein of unknown function (DUF3509)	C246T	-
Pchs_05361	*aroC*	chorismate synthase	G95A	-
Pchs_05364	*prmB*	[LSU ribosomal protein L3P]-glutamine N5-methyltransferase	G661A	E221K
Pchs_05365		Nicotinamidase-related amidase	C199T, C238T	H67Y, P80S
Pchs_05368	*folE2*	GTP cyclohydrolase I	C355T	R119C
Pchs_05370	*gst*	glutathione S-transferase	G151A	G51S
Pchs_05380		Uncharacterized membrane protein	C387T	-
Pchs_05384		hypothetical protein	C605T	A202V
Pchs_05408		Uncharacterized conserved protein	C453A	N151K
Pchs_05508		hypothetical protein	G67A	V23L
Pchs_05524		hypothetical protein	C60T	-

Pchs_05525	*astB*	succinylarginine dihydrolase	C1020T	-
Pchs_05644		hemoglobin/transferrin/lactoferrin receptor protein	G2188A	A730T
Pchs_05712		methyl-accepting chemotaxis sensory transducer with Cache sensor	C719T	T240I
Pchs_05821		Signal transduction histidine kinase	G1774A	A592T
Pchs_05833		transcriptional regulator, LuxR family	G254C	G85A
Pchs_01728		Sel1 repeat-containing protein	G932A	-
Pchs_02029		Beta-galactosidase	G741A	W247TC
Pchs_02056		ATP-dependent helicase HepA	C387T	-
Pchs_02081		MFS transporter, DHA1 family, arabinose polymer transporter	C433T	P145S
Pchs_00576	*pgk*	phosphoglycerate kinase	C488T	A163V
Pchs_05069		hypothetical protein	C203T	P68L
Pchs_05126		hypothetical protein	G467A	W156TC
Pchs_05132		Zn-binding Pro-Ala-Ala-Arg (PAAR) domain-containing protein, incolved in TypeVI secretion	C172T	L58F
Pchs_05134		quinohemoprotein amine dehydrogenase, alpha subunit	C813T	-
Pchs_05147		Nicotinamidase-related amidase	G187A	V63I
Pchs_05152	*pvdI*	non-ribosomal peptide synthase domain TIGR01720/amino acid adenylation domain-containing protein	C505T	-
Pchs_04310		Response regulator containing a CheY-like receiver domain and an HTH DNA-binding domain	G19A, A228C, C448T	A7T, E76D, H150Y
Pchs_06597		NAD(P)-dependent dehydrogenase, short-chain alcohol dehydrogenase family	G243A, G248A, A291G, G587A	-
Pchs_06598		FAD/FMN-containing dehydrogenase	T678C	-
Pchs_00904		LSU ribosomal protein L9P	G425A	R142H
Pchs_01095		3-hydroxybutyryl-CoA dehydrogenase	C799G	R267G
Pchs_01388	*lapA*	surface adhesion protein	T12505A, C12516T	S4169T
Pchs_03375		gamma-glutamylputrescine oxidase	G888A	-
Pchs_03410		sodium/proton antiporter, CPA1 family (TC 2.A.36)	C745T	-
Pchs_03532		ABC-type uncharacterized transport system involved in gliding motility, auxiliary component	C549T	-
Pchs_03535		hypothetical protein	G912A	-

Pchs_03580	*lspL*	UDP-glucuronate 4-epimerase	G1024A	A342T
Pchs_03773		hypothetical protein	C221T	T74I
Pchs_03775	*lhgO*	L-2-hydroxyglutarate oxidase	C45T	-
Pchs_03878	*lysP*	lysine:proton symporter, AAT family (TC 2.A.3.1.2)	C957T	-
Pchs_03920	*rocE*	arginine:proton symporter, AAT family (TC 2.A.3.1.11)	G369A	W123TC
Pchs_03967		TRAP transporter, DctM subunit	G971A	G324D
Pchs_04127		transposase, IS4 family	G347A	R116H
Pchs_04140		N-carbamoyl-L-amino-acid hydrolase	C799T	P267S
Pchs_04148		putative copper resistance protein D	G687A	-
Pchs_04161		Uncharacterized conserved protein, Alpha-E superfamily	C183T	-
Pchs_04167		Pimeloyl-ACP methyl ester carboxylesterase	G692A	W231TC
Pchs_04182	*alkC*	3-methyladenine DNA glycosylase AlkC	G788A	G263D
Pchs_04183		conserved hypothetical protein	G152A	R51Q
Pchs_04190		epralysin. Metallo peptidase. MEROPS family M10B	C725T	P242L
Pchs_04199		transcriptional regulator, LysR family	G141A	-
Pchs_04208		outer membrane autotransporter barrel domain-containing protein	G3392A	-
Pchs_04216		NAD(P)H dehydrogenase (quinone)	C402T	-
Pchs_04229		membrane fusion protein, multidrug efflux system	G916A, G941A	V306M, S314N
Pchs_04232		transcriptional regulator, AraC family with amidase-like domain	C858T	-
Pchs_04234		putative protease	C1935T	-
Pchs_04247		DNA-binding transcriptional regulator, LysR family	C633T	-
Pchs_04255		hypothetical protein	C50T	A17V
Pchs_04262	*rhtA*	Threonine/homoserine efflux transporter RhtA	G814A	D272N
Pchs_04273		ribosomal protein S12 methylthiotransferase accessory factor	G1279A	V427M
Pchs_04274		tRNA (adenine22-N1)-methyltransferase	G241A	A81T
Pchs_00734	*icmF*	type VI secretion system protein	G2935A	A979T
Pchs_01454		Fructose-2,6-bisphosphatase	G374A	G125D
Pchs_04312		VCBS repeat-containing protein	C604T, G620A, C670A	R207K, N220K, H224N
Pchs_04325		hypothetical protein	A284C	D95A

Pchs_04406		transcriptional regulator, LuxR family	G205A	G69S
Pchs_04407	*ofaA*	arthrofactin-type cyclic lipopeptide synthetase A	T5677G	V1893G

Supplementary Table S2. All 452 proteins expressed differentially in mutant P3.

[a] Expression fold change between two strains (mutant P3/control HT66), ratio > 1.5 represented upregulation, ratio < 0.7 represented downregulation.

Protein description	Gene	IMG locus tag_HT66	NCBI GI_HT66	Ratio[a]
Predicted acyl-CoA transferases/carnitine dehydratase		Pchs_00002	515074065	2.65
Acyl-CoA dehydrogenases	*gcdH*	Pchs_00003	647491551	2.19
NAD/NADP transhydrogenase alpha subunit	*pntAA*	Pchs_00005	515074066	0.67
Uncharacterized conserved small protein		Pchs_00009	496332375	0.66
cytochrome c oxidase, subunit II	*ctaC*	Pchs_00060	496337085	0.67
hypothetical protein [Pseudomonas chlororaphis]		Pchs_00079	647491723	0.45
tryptophan synthase, beta chain (EC 4.2.1.20)	*trpB*	Pchs_00083	496337064	0.30
tryptophan synthase, alpha chain (EC 4.2.1.20)	*trpA*	Pchs_00084	647491730	0.54
chromosomal replication initiator protein DnaA	*dnaA*	Pchs_00118	647491804	0.66
glucose-inhibited division protein A	*gidA*	Pchs_00122	647491816	0.67
chromosome segregation DNA-binding protein	*parB*	Pchs_00125	647491821	0.63
Predicted dehydrogenases and related proteins		Pchs_00143	647491911	0.39
Cyclopropane fatty acid synthase and related methyltransferases		Pchs_00181	647492002	0.50
transcriptional regulator, RpiR family		Pchs_00207	495202810	0.66
phosphate ABC transporter substrate-binding protein, PhoT family (TC 3.A.1.7.1)	*pstS*	Pchs_00239	495203739	0.37
ABC-type uncharacterized transport system, permease component	*pstC*	Pchs_00240	647492140	0.54
phosphate ABC transporter, permease protein PstA	*pstA*	Pchs_00241	496342427	0.58
phosphate ABC transporter ATP-binding protein, PhoT family (TC 3.A.1.7.1)	*pstB*	Pchs_00242	496342426	0.54
phosphate uptake regulator, PhoU	*phoU*	Pchs_00243	495203747	0.65
phosphate regulon transcriptional regulatory protein PhoB	*phoB*	Pchs_00249	495171678	0.65
Uncharacterized protein conserved in bacteria		Pchs_00250	647492159	0.59
type VI secretion protein, VC_A0107 family	*tssB*	Pchs_00257	495205505	2.09
type VI secretion protein, EvpB/VC_A0108 family	*tssC*	Pchs_00258	495205504	2.22
type VI secretion protein, VC_A0110 family	*tssF*	Pchs_00265	647492201	2.50
type VI secretion ATPase, ClpV1 family	*clpV*	Pchs_00267	647492206	1.77
type VI secretion protein, VC_A0114 family	*tssK*	Pchs_00272	495205486	1.74
type IV / VI secretion system protein, DotU family	*tssL*	Pchs_00273	647492220	1.65
type VI secretion protein IcmF	*tssM*	Pchs_00274	647492224	1.99
Transcriptional regulator, oxyR LysR family transcriptional regulator, hydrogen	*oxyR*	Pchs_00285	647492247	0.67

peroxide-inducible genes activator				
outer membrane transport energization protein ExbB (TC 2.C.1.1.1)	*exbB 1*	Pchs_00288	647492252	1.95
rpoZ DNA-directed RNA polymerase subunit omega (EC 2.7.7.6)	*rpoZ*	Pchs_00293	495196339	0.66
hypothetical protein [Pseudomonas]			495196536	0.60
Predicted enzyme of the cupin superfamily		Pchs_00308	647492291	0.50
Transcriptional regulators containing a DNA-binding HTH domain and an aminotransferase domain (MocR family) and their eukaryotic orthologs		Pchs_00312	647492301	0.39
Glycine/D-amino acid oxidases (deaminating)		Pchs_00317	647492310	3.53
Cytochrome c5		Pchs_00321	496336996	0.64
nitrogen regulatory protein P-II family	*glnK*	Pchs_00342	488618959	2.25
adenylate cyclase	*cyaA*	Pchs_00356	647492483	4.64
amino acid ABC transporter substrate-binding protein, PAAT family (TC 3.A.1.3.-)		Pchs_00385	647492551	5.6
hypothetical protein		Pchs_00414	647492615	0.29
Domain of unknown function (DUF4399)			496342187	0.60
Phosphoglycerate dehydrogenase and related dehydrogenases	*serA*	Pchs_00455	495208070	0.43
IAP aminopeptidase. Metallo peptidase. MEROPS family M28C	*papB*	Pchs_00460	647492691	5.13
Protein of unknown function (DUF2868)		Pchs_00480	647492739	5.06
hypothetical protein		Pchs_00492	647492777	1.80
NAD-dependent aldehyde dehydrogenases	*calB*	Pchs_00493	647492779	1.56
Domain of unknown function (DUF4426)		Pchs_00514	495196403	0.61
Uncharacterized conserved protein		Pchs_00557	495207754	1.53
adenosylhomocysteinase (EC 3.3.1.1)	*ahcY*	Pchs_00562	496336815	0.60
diguanylate cyclase (GGDEF) domain		Pchs_00581	647492989	0.32
choline dehydrogenase (EC 1.1.99.1)	*betA*	Pchs_00592	647493019	0.62
betaine aldehyde dehydrogenase (EC 1.2.1.8)	*betB*	Pchs_00593	647493021	0.49
Uncharacterized protein conserved in bacteria		Pchs_00613	647493058	0.63
Fe-S oxidoreductase		Pchs_00618	647493074	0.45
TonB-dependent siderophore receptor	*fhuA*	Pchs_00644	647493125	15.86
Glycine cleavage system regulatory protein		Pchs_00651	495201266	0.55
Acyl-CoA dehydrogenases		Pchs_00663	647493174	0.63
lysine 2-monooxygenase (EC 1.13.12.2)		Pchs_00681	647493223	1.77
Alcohol dehydrogenase, class IV		Pchs_00715	647493337	1.72
anthranilate synthase component I, non-proteobacterial lineages	*trpE*	Pchs_00718	647493343	0.67
nucleoside ABC transporter ATP-binding protein		Pchs_00761	647494590	4.34
nucleoside-binding protein		Pchs_00770	647494616	4.34

hypothetical protein [Pseudomonas chlororaphis]		Pchs_00780	647494638	0.60
Hemolysin activation/secretion protein		Pchs_00789	647494658	1.64
Uncharacterized protein conserved in bacteria		Pchs_00842	647494792	1.73
TonB-dependent copper receptor	*oprC*	Pchs_00844	647494796	1.99
urea ABC transporter, ATP-binding protein UrtD	*urtD*	Pchs_00853	647494818	2.48
urease. Metallo peptidase. MEROPS family M38 ureC urease subunit alpha [EC:3.5.1.5]	*ureC*	Pchs_00860	647494842	1.83
Urease accessory protein UreE	*ureE*	Pchs_00876	647494888	2.64
urease accessory protein UreG	*ureG*	Pchs_00878	496337543	2.79
Short-chain alcohol dehydrogenase of unknown specificity		Pchs_00882	495200436	0.51
amino acid/amide ABC transporter ATP-binding protein 2, HAAT family (TC 3.A.1.4.-)	*livF*	Pchs_00883	496337539	3.21
amino acid/amide ABC transporter ATP-binding protein 1, HAAT family (TC 3.A.1.4.-)	*livG*	Pchs_00884	565889081	3.35
amino acid/amide ABC transporter substrate-binding protein, HAAT family (TC 3.A.1.4.-)	*livJ*	Pchs_00887	496337535	3.50
azurin	*azu*	Pchs_00893	496337529	0.55
ADP-ribose pyrophosphatase		Pchs_00897	647494936	1.73
Uncharacterized protein conserved in bacteria		Pchs_00900	647494946	2.44
Oligoribonuclease (3'->5' exoribonuclease)	*orn*	Pchs_00928	647495064	0.65
Uncharacterized protein conserved in bacteria		Pchs_00937	647495082	0.70
thioredoxin		Pchs_00994	647495226	0.66
Uncharacterized protein conserved in bacteria		Pchs_01042	496332636	0.51
poly(hydroxyalkanoate) granule-associated protein		Pchs_01047	647495374	0.60
poly(hydroxyalkanoate) granule-associated protein	*PhaI*	Pchs_01048	647495377	0.55
Methyl-accepting chemotaxis protein		Pchs_01059	647495401	0.18
urocanate hydratase (EC 4.2.1.49)	*hutU*	Pchs_01076	515074600	1.67
histidine utilization repressor, proteobacterial	*hutC*	Pchs_01078	647495443	0.63
formimidoylglutamate deiminase (EC 3.5.3.13)	*hutF*	Pchs_01079	647495445	0.53
glycogen/starch/alpha-glucan phosphorylases	*glgP*	Pchs_01085	647495459	3.25
L-glutamine synthetase (EC 6.3.1.2)	*glnA*	Pchs_01089	647495467	3.05
nitrogen regulation protein NR(I)	*glnG*	Pchs_01102	647495504	4.04
Membrane-bound metallopeptidase		Pchs_01110	647495517	0.65
amino acid ABC transporter substrate-binding protein, PAAT family (TC 3.A.1.3.-)		Pchs_01130	496337335	1.62
hypothetical protein		Pchs_01164	647495647	0.43
hypothetical protein [Pseudomonas chlororaphis]		Pchs_01166	647495649	0.64
DNA-methyltransferase (dcm)		Pchs_01187	647495708	0.63
ADP-ribose pyrophosphatase	*nudE*	Pchs_01218	495206934	0.64
BON domain/LysM domain		Pchs_01220	496332538	0.52
L-cystine ABC transporter ATP-binding		Pchs_01264	647495903	0.64

protein/Diaminopimelate ABC transporter ATP-binding protein				
RND family efflux transporter, MFP subunit		Pchs_01274	647495933	1.60
ABC-type metal ion transport system, periplasmic component/surface antigen		Pchs_01282	647495948	0.59
type VI secretion system effector, Hcp1 family	*hcpA*	Pchs_01292	495200970	1.59
5-aminovalerate transaminase (EC 2.6.1.48)	*gabT*	Pchs_01325	496332446	1.58
hypothetical protein [Pseudomonas chlororaphis]		Pchs_01330	647496042	2.14
Aspartyl/asparaginyl beta-hydroxylase and related dioxygenases		Pchs_01362	647496100	0.67
type I secretion C-terminal target domain (VC_A0849 subclass)	*lapA*	Pchs_01388	647496163	1.72
pyocin immunity protein [Pseudomonas chlororaphis]			647496769	0.66
methylmalonate-semialdehyde dehydrogenase [acylating] (EC 1.2.1.27)	*mmsA-2*	Pchs_01422	647499383	2.34
3-hydroxyisobutyrate dehydrogenase (EC 1.1.1.31)	*mmsB*	Pchs_01423	647499387	1.70
anthranilate dioxygenase, alpha subunit	*antA*	Pchs_01433	647499410	1.51
Kynureninase (EC 3.7.1.3)	*kynU*	Pchs_01436	647499417	1.88
gamma-glutamyltransferase 1 (EC:2.3.2.2). Threonine peptidase. MEROPS family T03	*ggt*	Pchs_01444	647499437	2.58
Spermidine/putrescine-binding periplasmic protein	*potF*	Pchs_01465	647499488	1.54
FAD/FMN-containing dehydrogenases		Pchs_01478	647499515	1.64
transcriptional regulator, GntR family	*pdhR*	Pchs_01483	496337740	0.66
Fe^{2+}/Zn^{2+} uptake regulation proteins	*fur*	Pchs_01489	495196050	0.66
carbamoyl-phosphate synthase small subunit	*carA*	Pchs_01497	647499558	0.64
transcription elongation factor GreA	*greA*	Pchs_01499	647499565	0.58
Uncharacterized protein conserved in bacteria		Pchs_01509	496332949	0.50
tRNA pseudouridine synthase B (EC 4.2.1.70)	*truB*	Pchs_01513	495196094	0.67
acetoacetyl-CoA synthase	*acsA_1*	Pchs_01546	647499663	2.42
3-hydroxybutyrate dehydrogenase	*bdhA*	Pchs_01547	647499665	4.50
ppiC Parvulin-like peptidyl-prolyl isomerase	*ppiC_1*	Pchs_01554	495199663	0.68
thioredoxin [Pseudomonas chlororaphis]			647490025	0.46
TonB-dependent siderophore receptor	*piuA*	Pchs_01589	647490308	8.56
Uncharacterized iron-regulated protein		Pchs_01590	647490310	2.18
Diaminopimelate decarboxylase	*speC*	Pchs_01592	647490314	1.61
ABC-type dipeptide transport system, periplasmic component	*dppA_1*	Pchs_01603	647490337	1.78
outer membrane porin, OprD family	*OprD*	Pchs_01604	647490339	3.10
ABC-type dipeptide transport system, periplasmic	*dppA*	Pchs_01605	647490341	2.02

component	*_2*			
aspartyl/glutamyl-tRNA (Asn/Gln) amidotransferase subunit A (EC 6.3.5.-)	*gatA*	Pchs_01624	647490384	0.61
fumarase, class II (EC 4.2.1.2)	*fumC_1*	Pchs_01637	647490465	5.57
ABC-type transport system involved in resistance to organic solvents, auxiliary component, ttg2 putative toluene tolerance protein	*ttg2D*	Pchs_01654	647490488	0.69
Flavodoxins		Pchs_01682	515071874	0.61
TonB-dependent siderophore receptor	*fecA*	Pchs_01712	647490614	1.67
Domain of unknown function (DUF4123)		Pchs_01732	647490661	0.53
hypothetical protein [Pseudomonas]		Pchs_01733	495205272	0.52
hypothetical protein [Pseudomonas chlororaphis]		Pchs_01734	647490664	0.43
Predicted ATPase related to phosphate starvation-inducible protein PhoH		Pchs_01752	496337918	2.41
Predicted esterase	*estB*	Pchs_01761	647490726	0.57
amino acid ABC transporter substrate-binding protein, PAAT family (TC 3.A.1.3.-)	*aapJ*	Pchs_01762	647490729	7.84
amino acid ABC transporter membrane protein 1, PAAT family (TC 3.A.1.3.-)	*aapQ*	Pchs_01763	647490732	2.49
amino acid ABC transporter ATP-binding protein, PAAT family (TC 3.A.1.3.-)	*aapP*	Pchs_01765	496337929	4.70
NADH:flavin oxidoreductases, Old Yellow Enzyme family	*xenA*	Pchs_01827	647490887	0.56
Chemotaxis protein histidine kinase and related kinases		Pchs_01847	647490936	0.57
bacterial peptide chain release factor 2 (bRF-2)	*prfB*	Pchs_01850	515075469	0.57
periplasmic chaperone for outer membrane proteins Skp		Pchs_01900	495199277	0.60
acetyl-CoA carboxylase carboxyltransferase subunit alpha	*accA*	Pchs_01907	495199263	0.57
Negative regulator of beta-lactamase expression	*amiD*	Pchs_01929	647491106	0.19
Flavodoxin reductases (ferredoxin-NADPH reductases) family	*fpr_1*	Pchs_01981	495199142	0.66
Acetyltransferases		Pchs_01986	647491222	0.62
Protein of unknown function, DUF481		Pchs_02006	647491261	0.64
amino acid ABC transporter ATP-binding protein, PAAT family (TC 3.A.1.3.-)	*hisP*	Pchs_02011	647491334	1.58
amino acid ABC transporter substrate-binding protein, PAAT family (TC 3.A.1.3.-)	*hisJ*	Pchs_02015	496338179	1.65
hypothetical protein [Pseudomonas chlororaphis]			496338965	2.14
Pyridoxamine 5'-phosphate oxidase (EC 1.4.3.5)	*pdxH*	Pchs_02032	647491381	0.57
L-leucine-binding protein/L-isoleucine-binding	*braC*	Pchs_02049	647491415	1.66

protein/L-valine-binding protein				
3-hydroxyisobutyrate dehydrogenase and related beta-hydroxyacid dehydrogenases		Pchs_02052	647491422	0.68
NADH:flavin oxidoreductases, Old Yellow Enzyme family	*xenB*	Pchs_02080	647491477	0.57
Phosphatidylserine/phosphatidylglycerophosphate/cardiolipin synthases and related enzymes		Pchs_02094	647491511	1.77
Dihydrodipicolinate synthase/N-acetylneuraminate lyase	*dapA*	Pchs_02172	647483598	0.40
Inclusion body protein		Pchs_02183	647483633	0.52
Protein of unknown function (DUF2971)		Pchs_02197	647483663	0.49
Acetyltransferases, including N-acetylases of ribosomal proteins		Pchs_02198	647483665	0.41
hypothetical protein [Pseudomonas chlororaphis]		Pchs_02220	647486676	0.68
Predicted hydrolases or acyltransferases (alpha/beta hydrolase superfamily)		Pchs_02231	496338294	0.25
periplasmic serine protease, Do/DeqQ family	*mucD*	Pchs_02253	647486746	0.62
DNA gyrase inhibitor		Pchs_02349	647486984	0.56
type I secretion membrane fusion protein, HlyD family	*lapC*	Pchs_02366	647487032	2.19
type I secretion system ATPase, LssB family	*lapB*	Pchs_02367	647487034	2.33
type I secretion outer membrane protein, TolC family	*lapE*	Pchs_02368	647487037	2.90
VCBS repeat/type I secretion C-terminal target domain (VC_A0849 subclass)	*lapA*	Pchs_02369	647487039	2.66
leucyl aminopeptidase [Pseudomonas chlororaphis]		Pchs_02398	647487111	0.49
Flagellar basal-body P-ring protein	*flgI*	Pchs_02412	647487141	0.47
CDP-glucose 4,6-dehydratase	*rfbG*	Pchs_02418	647487156	0.29
UDP-N-acetylglucosamine 4,6-dehydratase	*wbjB*	Pchs_02425	647487176	0.57
pseudaminic acid CMP-transferase	*pseF*	Pchs_02427	647487181	0.63
3-oxoacyl-[acyl-carrier-protein] synthase III	*fabH_2*	Pchs_02430	647487188	0.53
fliC Flagellin and related hook-associated proteins	*fliC*	Pchs_02433	647487196	0.11
flagellar biosynthetic protein FliS	*fliS*	Pchs_02436	495197853	0.32
flagellar motor switch protein FliG	*fliG*	Pchs_02443	495197841	0.40
FOG: HPt domain		Pchs_02449	647487222	0.52
ATPases involved in chromosome partitioning	*fleN*	Pchs_02461	647487254	0.65
RNA polymerase, sigma 28 subunit, SigD/FliA/WhiG fliA RNA polymerase sigma factor for flagellar operon FliA	*fliA*	Pchs_02462	495197716	0.49
Response regulator containing CheY-like receiver, AAA-type ATPase, and DNA-binding domains	*cheY*	Pchs_02463	498142783	0.19

Chemotaxis protein	*cheZ*	Pchs_02464	496333571	0.31
Chemotaxis protein histidine kinase and related kinases cheA	*cheA*	Pchs_02465	647487264	0.22
Chemotaxis response regulator containing a CheY-like receiver domain and a methylesterase domain	*cheB*	Pchs_02466	647487268	0.36
ATPases involved in chromosome partitioning		Pchs_02469	495197709	0.52
Chemotaxis signal transduction protein	*cheW*	Pchs_02471	495197707	0.15
GDSL-like Lipase/Acylhydrolase family		Pchs_02502	647487349	0.56
hypothetical protein		Pchs_02503	647487351	0.25
Predicted carbamoyl transferase, NodU family		Pchs_02506	647487358	0.33
Methionyl-tRNA formyltransferase		Pchs_02509	647487362	0.50
citrate synthase (EC 2.3.3.1)	*gltA*	Pchs_02520	496338587	3.05
Methyl-accepting chemotaxis protein		Pchs_02545	647487424	0.49
hypothetical protein		Pchs_02623	647483845	0.47
Nucleoside-binding outer membrane protein	*Tsx*	Pchs_02673	647483979	1.94
Purine nucleoside permease		Pchs_02674	647483981	2.56
L-arginine-binding protein/L-ornithine-binding protein	*aotJ*	Pchs_02692	496339016	1.67
Acetylornithine deacetylase/Succinyl-diaminopimelate desuccinylase and related deacylases	*argE 2*	Pchs_02707	647484051	1.70
hypothetical protein		Pchs_02712	647484063	0.34
TonB-dependent siderophore receptor		Pchs_02718	647484077	1.65
Acyl-CoA dehydrogenases		Pchs_02730	647484099	0.65
haloacid dehalogenase superfamily, subfamily IA, variant 3 with third motif having DD or ED		Pchs_02732	647484104	0.64
Domain of unknown function (DUF1842)/Domain of unknown function (DUF1843)		Pchs_02737	647484112	2.27
Domain of unknown function (DUF1842)/Domain of unknown function (DUF1843)		Pchs_02738	647484114	1.9
Y_X(10)_GDL-associated radical SAM protein	*nirJ*	Pchs_02739	647484117	1.91
beta-ketoacyl-acyl-carrier-protein synthase II	*fabF _2*	Pchs_02742	647484125	0.41
threonyl-tRNA synthetase (EC 6.1.1.3)	*thrS*	Pchs_02767	647484335	0.57
chitinase family 18 (EC:3.2.1.14)	*chiC*	Pchs_02788	647484393	0.12
chitin-binding protein		Pchs_02789	647484396	0.08
acetyl-CoA carboxylase carboxyltransferase subunit alpha	*accD*	Pchs_02808	647484424	0.67
transcription-repair coupling factor	*mfd*	Pchs_02867	647484573	0.67
3-ketoacyl-CoA thiolase (EC 2.3.1.16)	*fadA*	Pchs_02879	496338784	0.59
short chain enoyl-CoA hydratase (EC 4.2.1.17) /3-hydroxyacyl-CoA dehydrogenase (EC 1.1.1.35)	*fadB*	Pchs_02880	647484600	0.62

methyl-accepting chemotaxis sensory transducer with Pas/Pac sensor	*aer-2*	Pchs_02893	647484628	0.45
cAMP-binding proteins - catabolite gene activator and regulatory subunit of cAMP-dependent protein kinases	*Anr*	Pchs_02911	496338758	0.43
DNA ligase, NAD-dependent	*ligA*	Pchs_02927	647484695	0.62
ABC-type nitrate/sulfonate/bicarbonate transport systems, periplasmic components	*nrtA*	Pchs_02944	647484724	2.81
assimilatory nitrite reductase (NAD(P)H) large subunit precursor (EC 1.7.1.4)	*nasR*	Pchs_02949	647484728	3.23
RNA polymerase sigma factor, sigma-70 family	*sigX*	Pchs_02954	647484734	0.64
RraA famliy		Pchs_02958	495202726	0.60
Hydantoin racemase		Pchs_02976	647484766	1.64
ribosomal large subunit pseudouridine synthase C (EC 5.4.99.24)	*rluC*	Pchs_03044	647484926	0.68
Aerobic-type carbon monoxide dehydrogenase, large subunit CoxL/CutL homologs	*iorB*	Pchs_03049	647484937	0.65
Methyltransferase domain		Pchs_03081	647485012	1.53
Terpene synthase family, metal binding domain		Pchs_03082	647485014	3.23
hypothetical protein [Pseudomonas chlororaphis]			647485471	0.66
hypothetical protein [Pseudomonas chlororaphis]			647485614	0.37
hypothetical protein [Pseudomonas chlororaphis]			647485694	0.22
hypothetical protein [Pseudomonas chlororaphis]			647485711	0.67
hypothetical protein [Pseudomonas chlororaphis]			647485737	0.61
hypothetical protein [Pseudomonas chlororaphis]			647485782	0.54
3-oxoacid CoA-transferase, A subunit ScoA	*scoA*	Pchs_03129	495195793	71.60
3-oxoacid CoA-transferase, B subunit ScoB	*scoB*	Pchs_03130	495195791	7.49
acetyl-CoA acetyltransferase (EC 2.3.1.9)	*atoB*	Pchs_03131	647487719	9.64
Uncharacterized conserved protein		Pchs_03157	496340180	0.63
molybdenum ABC transporter, periplasmic molybdate-binding protein	*modA*	Pchs_03162	647496278	1.55
4-hydroxyphenylpyruvate dioxygenase (EC 1.13.11.27)	*hpd*	Pchs_03189	515072615	1.50
transcriptional regulator, LysR family		Pchs_03193	647496340	0.64
Acyl-CoA synthetases (AMP-forming)/AMP-acid ligases II		Pchs_03195	647496344	0.64
2, 4-dihydroxyhept-2-enedioate aldolase (EC 4.1.2.-)	*hpaI*	Pchs_03199	515072622	1.75
Amidases related to nicotinamidase		Pchs_03238	647496421	0.68
Uncharacterized protein conserved in bacteria		Pchs_03280	647496507	0.15
chitinase family 18 (EC:3.2.1.14)	*chiC*	Pchs_03281	647496510	0.20
Acetyl-CoA acetyltransferase		Pchs_03284	647496514	1.53
outer membrane autotransporter barrel	*pspA*	Pchs_03329	647496602	2.95

domain/autotransporter-associated beta strand repeat				
outer membrane autotransporter barrel domain/autotransporter-associated beta strand repeat	*pspB*	Pchs_03330	647496604	1.66
Uncharacterized conserved protein		Pchs_03366	647496683	0.41
TonB-dependent siderophore receptor		Pchs_03383	647496719	4.50
diaminobutyrate aminotransferase apoenzyme (EC 2.6.1.76)	*acsF*	Pchs_03384	647496722	13.41
Siderophore synthetase component (Achromobactin biosynthetic protein AcsD)	*acsD*	Pchs_03385	647496724	4.28
Siderophore synthetase component	*acsA*	Pchs_03390	647496739	7.35
ABC-type cobalamin/Fe3+-siderophores transport systems, ATPase components	*fepC*	Pchs_03394	647496750	2.85
Demethylmenaquinone methyltransferase		Pchs_03396	647496754	2.32
hypothetical protein		Pchs_03400	647496767	0.65
diguanylate cyclase (GGDEF) domain	*wspR*	Pchs_03415	647496806	0.63
Choline dehydrogenase and related flavoproteins	*betA*	Pchs_03454	647496910	1.50
Phosphatidylserine/phosphatidylglycerophosphate/cardiolipin synthases and related enzymes		Pchs_03455	647496912	0.64
5'-nucleotidase, lipoprotein e (P4) family		Pchs_03462	647496922	1.75
ABC-type dipeptide transport system, periplasmic component		Pchs_03467	647496930	1.54
hypothetical protein		Pchs_03487	647496973	0.43
hypothetical protein [Pseudomonas chlororaphis]		Pchs_03495	565883881	0.14
Amidases related to nicotinamidase Isochorismatase-like		Pchs_03513	647497037	0.60
Glycosyltransferases involved in cell wall biogenesis	*arnC*	Pchs_03538	647497098	0.62
nucleotide sugar dehydrogenase		Pchs_03544	496339793	0.66
Acyl-CoA dehydrogenases		Pchs_03562	647497157	1.95
short chain enoyl-CoA hydratase (EC 4.2.1.17)	*fadB 1x*	Pchs_03563	647497158	1.56
acetyl-CoA acetyltransferases		Pchs_03565	647497160	1.57
Acyl-coenzyme A synthetases/AMP-(fatty) acid ligases		Pchs_03567	647497165	2.74
TonB-dependent siderophore receptor		Pchs_03569	647497170	3.11
Serine protease inhibitor ecotin	*eco*	Pchs_03604	647497248	0.54
Glucose/sorbosone dehydrogenases	*ylil*	Pchs_03613	647497267	1.60
phenylacetic acid degradation protein paaN	*paaN*	Pchs_03653	647497358	1.90
Protein related to penicillin acylase	*pvdQ*	Pchs_03685	647497454	3.06
maltooligosyl trehalose hydrolase (EC 3.2.1.141)	*treZ*	Pchs_03700	647497476	1.85
glycogen debranching enzyme GlgX	*glgX*	Pchs_03704	647497485	2.12
Predicted hydrolases or acyltransferases (alpha/beta hydrolase superfamily)		Pchs_03707	647497493	2.62

Transcriptional regulators containing a DNA-binding HTH domain		Pchs_03713	496339630	0.43
transcriptional regulator, LysR family		Pchs_03732	647497545	0.53
Parvulin-like peptidyl-prolyl isomerase		Pchs_03733	647497547	0.67
Putative bacterial sensory transduction regulator		Pchs_03734	647497550	0.61
hypothetical protein [Pseudomonas chlororaphis]		Pchs_03746	647497576	0.34
FecR family protein		Pchs_03763	647497614	0.64
transcriptional regulator, GntR family		Pchs_03774	647497639	0.65
Predicted exporters of the RND superfamily		Pchs_03799	647497686	1.76
Uncharacterized protein conserved in bacteria		Pchs_03804	495196115	0.57
diguanylate cyclase (GGDEF) domain		Pchs_03830	647497756	1.95
Aerobic-type carbon monoxide dehydrogenase, large subunit CoxL/CutL homologs	*iorB*	Pchs_03832	647497761	1.62
phage shock protein A (PspA) family protein		Pchs_03845	565884938	0.55
Ribonuclease I		Pchs_03860	647497816	0.40
Uncharacterized paraquat-inducible protein B		Pchs_03876	647497849	0.51
hypothetical protein [Pseudomonas chlororaphis]		Pchs_03904	496339451	0.67
Predicted hydrolases or acyltransferases (alpha/beta hydrolase superfamily)		Pchs_03930	647497983	1.51
Protein of unknown function (DUF3616)		Pchs_03957	647498051	1.75
hypothetical protein [Pseudomonas chlororaphis]		Pchs_03971	647498082	2.17
Common central domain of tyrosinase		Pchs_04012	647498174	1.92
Sortase and related acyltransferases		Pchs_04031	647498220	2.33
aconitase (EC 4.2.1.3)	*acnB*	Pchs_04041	647498241	0.60
hypothetical protein [Pseudomonas chlororaphis]		Pchs_04125	647498419	0.43
transposase, IS4 family		Pchs_04127	647498421	0.65
amidase, hydantoinase/carbamoylase family		Pchs_04140	647498458	2.07
Pyruvate/2-oxoglutarate dehydrogenase complex, dehydrogenase (E1) component, eukaryotic type, beta subunit	*bkdA_2*	Pchs_04155	647498492	2.27
branched-chain alpha-keto acid dehydrogenase E2 component (EC 2.3.1.168)	*bkdB*	Pchs_04156	647498496	2.21
dihydrolipoamide dehydrogenase	*bkdC*	Pchs_04157	647498498	2.08
Uncharacterized conserved protein		Pchs_04162	495200274	3.56
Protein of unknown function (DUF2599)		Pchs_04188	647498598	0.18
Protein of unknown function (DUF2599)		Pchs_04189	647498601	0.37
Domain of unknown function (DUF4174)		Pchs_04209	647498751	0.60
Dienelactone hydrolase and related enzymes		Pchs_04245	647498852	0.64
Phosphatidylserine decarboxylase	*psd*	Pchs_04246	647498854	0.62
Predicted metal-dependent hydrolase with the TIM-barrel fold		Pchs_04356	496340404	0.66
type VI secretion system effector, Hcp1 family	*hcp1*	Pchs_04376	496335162	2.11
type VI secretion protein, VC_A0114 family	*tssK*	Pchs_04382	647500000	1.53

Predicted outer membrane lipoprotein	*tagQ*	Pchs_04391	647500023	0.59
hypothetical protein [Pseudomonas chlororaphis]		Pchs_04404	647500060	0.48
amino acid adenylation domain	*ofaB*	Pchs_04408	647500074	0.36
Dihydrodipicolinate synthase/N-acetylneuraminate lyase		Pchs_04427	647500141	0.68
The Hydrophobe/Amphiphile Efflux-1 (HAE1) Family	*mexF*	Pchs_04494	647500323	1.83
Serine/threonine protein kinase	*ppkA*	Pchs_04504	647500351	0.65
hypothetical protein [Pseudomonas chlororaphis]		Pchs_04510	647500371	0.16
Uncharacterized protein conserved in bacteria		Pchs_04526	647500433	0.13
IscR-regulated protein YhgI	*nfuA*	Pchs_04578	495197073	0.61
DNA-directed DNA polymerase III (polc),dnaE2 error-prone DNA polymerase	*dnaE 2*	Pchs_04621	647485479	1.70
cyclic pyranopterin monophosphate synthase subunit MoaC (EC 4.1.99.18)	*moa C-2*	Pchs_04660	647485587	1.68
Hypothetical protein (DUF2513)		Pchs_04684	647485640	0.50
hypothetical protein [Pseudomonas chlororaphis]		Pchs_04744	647485792	0.65
Inclusion body protein		Pchs_04752	647485811	0.32
Response regulator containing a CheY-like receiver domain and an HD-GYP domain		Pchs_04761	647485834	0.64
PAS domain S-box/diguanylate cyclase (GGDEF) domain		Pchs_04762	647485837	0.59
malate:quinone-oxidoreductase	*mqoA*	Pchs_04769	647485851	0.52
periplasmic chaperone LolA	*lolA*	Pchs_04785	647485891	0.57
thioredoxin-disulfide reductase	*trxB2*	Pchs_04787	647485896	1.66
cold-shock DNA-binding protein family	*cspD*	Pchs_04793	496340597	0.42
NADH dehydrogenase subunit I (EC 1.6.5.3)	*nuoI*	Pchs_04812	489316425	0.65
carbohydrate ABC transporter substrate-binding protein, CUT1 family (TC 3.A.1.1.-)	*glpV*	Pchs_04820	647485965	8.16
Exodeoxyribonuclease III (EC 3.1.11.2)	*xth*	Pchs_04838	647486006	0.62
phosphate ABC transporter substrate-binding protein, PhoT family (TC 3.A.1.7.1)		Pchs_04840	647486011	0.63
lysine-arginine-ornithine-binding periplasmic protein arginine/ornithine transport system substrate-binding protein	*aotJ*	Pchs_04910	647486203	3.73
methyl-accepting chemotaxis sensory transducer with Cache sensor		2527289721	647486219	0.10
hypothetical protein [Pseudomonas chlororaphis]		Pchs_04947	647486301	0.39
TonB-dependent siderophore receptor		Pchs_04973	647486375	1.69
1,2-dihydroxycyclohexa-3,5-diene-1-carboxylate dehydrogenase (EC 1.3.1.25)	*benD*	Pchs_04994	647486427	5.45
catechol 1,2-dioxygenase, proteobacterial	*catA*	Pchs_04999	647486441	2.36
muconate cycloisomerase (EC 5.5.1.1)	*catB*	Pchs_05001	647486448	1.97

Beta-lactamase class C and other penicillin binding proteins	*amp C*	Pchs_05032	647486525	2.64
hypothetical protein [Pseudomonas chlororaphis]		Pchs_05036	647486536	0.59
Zn-dependent dipeptidase, microsomal dipeptidase homolog	*pvdM*	Pchs_05058	647486584	2.96
Uncharacterized conserved protein pvdO	*pvdO*	Pchs_05060	647486586	2.55
Rhs element Vgr protein	*pvdD*	Pchs_05064	647486599	1.805
type VI secretion system secreted protein VgrG	*vgrG*	Pchs_05080	647499197	0.69
Deacetylases, including yeast histone deacetylase and acetoin utilization protein	*aphA 2*	Pchs_05117	647493466	2.55
Predicted metal-dependent hydrolase with the TIM-barrel fold		Pchs_05126	647493486	0.21
amino acid adenylation domain	*pvdJ*	Pchs_05153	647493566	4.18
Cysteine sulfinate desulfinase/cysteine desulfurase and related enzymes		Pchs_05160	647487736	0.50
Uncharacterized protein conserved in bacteria		Pchs_05171	647487763	0.60
Methyltransferase domain		Pchs_05180	647487786	0.66
Thiol:disulfide interchange protein DsbC	*dsbG*	Pchs_05187	647487804	0.67
amino acid ABC transporter substrate-binding protein, PAAT family (TC 3.A.1.3.-)		Pchs_05196	647487824	0.56
Chemotaxis signal transduction protein		Pchs_05207	496335741	0.31
Enoyl-CoA hydratase/carnithine racemase		Pchs_05244	647487944	1.61
translation elongation factor P (EF-P)	*efp*	Pchs_05255	495206617	0.66
Transcriptional regulator	*cysB*	Pchs_05259	495206606	0.66
hypothetical protein [Pseudomonas chlororaphis]			647488032	0.53
Sec-independent protein translocase TatD (EC 3.1.21.-)	*tatD*	Pchs_05278	647488041	0.57
Osmosensitive K+ channel histidine kinase	*kdpD _1*	Pchs_05286	647488056	0.65
Uncharacterized conserved protein		Pchs_05293	647488072	3.36
Short-chain dehydrogenases of various substrate specificities		Pchs_05296	647488080	0.32
uraH, pucM, hiuH hydroxyisourate hydrolase	*uraH _1*	Pchs_05375	565885224	0.36
septum site-determining protein MinD	*minD*	Pchs_05388	496341044	0.69
cell division topological specificity factor MinE	*minE*	Pchs_05389	495203885	0.58
hypothetical protein [Pseudomonas chlororaphis]		Pchs_05446	647488412	0.25
(p)ppGpp synthetase, RelA/SpoT family	*relA*	Pchs_05460	496341096	1.51
Predicted ATPase of the PP-loop superfamily implicated in cell cycle control	*ttcA*	Pchs_05474	495207011	0.42
flagellar hook-basal body protein	*flgE*	Pchs_05488	647488512	0.33
Chemotaxis signal transduction protein	*cheV*	Pchs_05493	647488523	0.58
Predicted glycosyltransferase		Pchs_05497	647488533	0.36

Transcriptional regulator containing an amidase domain and an AraC-type DNA-binding HTH domain	*argA*	Pchs_05530	647488676	2.73
Ribonucleotide reductase, beta subunit		Pchs_05538	647488694	0.62
transcriptional regulator, TetR family		Pchs_05547	647488718	0.66
Uncharacterized protein conserved in bacteria		Pchs_05586	647488820	0.57
Aspartate/tyrosine/aromatic aminotransferase		Pchs_05588	495200347	2.09
Acyl dehydratase		Pchs_05619	495200297	0.64
porin, OprB family (TC 1.B.19)	*oprB*	Pchs_05630	647488916	1.56
TonB-dependent heme/hemoglobin receptor family protein/TonB-dependent hemoglobin/transferrin/lactoferrin receptor family protein	*phuR*	Pchs_05644	647488959	1.56
Heme oxygenase	*hemO*	Pchs_05645	647488963	0.45
hypothetical protein		Pchs_05655	647488987	0.64
Flp pilus assembly protein, ATPase CpaF	*cpaF*	Pchs_05669	647489032	1.78
Flp pilus assembly protein, ATPase CpaE	*cpaE*	Pchs_05670	647489034	1.53
Flp pilus assembly protein CpaB	*cpaB*	Pchs_05674	647489046	1.71
outer membrane porin, OprD family	*oprD*	Pchs_05700	647489116	1.67
methyl-accepting chemotaxis sensory transducer with Cache sensor	*pctA_1*	Pchs_05712	647489142	0.13
fumarase, class I, homodimeric (EC 4.2.1.2)		Pchs_05731	495206415	0.67
Superoxide dismutase	*sodB*	Pchs_05758	495204984	0.57
Protein affecting phage T7 exclusion by the F plasmid,fxsA UPF0716 protein FxsA		Pchs_05775	495207922	0.67
bacterioferritin	*bfr_1*	Pchs_05793	496341376	0.59
monothiol glutaredoxin, Grx4 family	*grxD*	Pchs_05794	495197636	0.49
Cys-tRNA(Pro) deacylase	*ybaK*	Pchs_05799	515073603	0.67
L-glutamate-binding protein/L-aspartate-binding protein	*gltI*	Pchs_05804	647489345	0.61
methionine synthase (B12-independent) (EC 2.1.1.14)	*metE*	Pchs_05826	647489417	0.34
transcriptional regulator, LysR family	*metR*	Pchs_05827	496341406	0.60
hypothetical protein		Pchs_05841	647489456	0.24
diguanylate cyclase/phosphodiesterase		Pchs_05855	647489490	3.01
ATP dependent PIM1 peptidase. Serine peptidase. MEROPS family S16,ATP-dependent Lon protease	*lon_2*	Pchs_05857	647489494	0.66
Maltoporin (phage lambda and maltose receptor)	*lamB*	Pchs_05866	647489516	0.68
inosine-5'-monophosphate dehydrogenase (EC 1.1.1.205)	*guaB*	Pchs_05873	647489531	0.63
Uncharacterized protein conserved in bacteria		Pchs_05888	647489562	0.67
Chaperone protein HscA	*hscA*	Pchs_05894	647489571	0.63

hypothetical protein [Pseudomonas chlororaphis]			647489597	1.80
Iron-binding apoprotein IscA	*iscA*	Pchs_05896	495204743	0.58
FeS assembly scaffold apoprotein IscU	*iscU*	Pchs_05897	495204742	0.62
cysteine desulfurase IscS	*iscS*	Pchs_05898	647489588	0.62
Archaeal fructose-1,6-bisphosphatase and related enzymes of inositol monophosphatase family	*suhB*	Pchs_05902	495204733	0.65
Uncharacterized conserved protein		Pchs_05927	647489639	0.41
outer membrane porin, OprD family		Pchs_05976	647489767	0.67
ubiquinol-cytochrome c reductase, iron-sulfur subunit	*petA*	Pchs_06004	495203673	0.67
Predicted oxidoreductases (related to aryl-alcohol dehydrogenases)	*tas*	Pchs_06007	647489831	0.49
Uncharacterized protein conserved in bacteria		Pchs_06013	495203647	0.48
hypothetical protein[Pseudomonas chlororaphis]		Pchs_06041	647489909	0.63
Phenazine biosynthesis protein A/B	*phzA*	Pchs_06091	647490047	5.60
Phenazine biosynthesis protein A/B	*phzB*	Pchs_06092	647490048	4.48
Isochorismate hydrolase	*phzD*	Pchs_06094	647490052	5.52
Anthranilate/para-aminobenzoate synthases component I	*phzE*	Pchs_06095	647490054	0.56
phenazine biosynthesis protein PhzF family	*phzF*	Pchs_06096	647490057	1.78
Pyridoxamine 5'-phosphate oxidase (EC 1.4.3.5)	*phzG*	Pchs_06097	647490058	2.58
phzH asparagine synthase (glutamine-hydrolyzing)	*phzH*	Pchs_06098	647490062	3.33
methyl-accepting chemotaxis sensory transducer with Pas/Pac sensor		Pchs_06122	647490123	0.47
Carbonic anhydrase	*cah_3*	Pchs_06146	496341688	0.56
Paraquat-inducible protein B		Pchs_06153	647485192	2.39
Uncharacterized low-complexity proteins		Pchs_06168	647487583	0.21
acetyl-coenzyme A synthetase (EC 6.2.1.1)	*acsA_2*	Pchs_06191	647487629	2.32
hypothetical protein [Pseudomonas chlororaphis]		Pchs_06205	647493612	0.40
mannose-1-phosphate guanylyltransferase /mannose-6-phosphate isomerase	*pslB*	Pchs_06207	647493618	2.76
Periplasmic protein involved in polysaccharide export	*pslD*	Pchs_06209	647493628	3.72
Uncharacterized protein involved in exopolysaccharide biosynthesis	*pslE*	Pchs_06210	647493630	2.43
Glycosyltransferase	*pslF*	Pchs_06211	647493633	1.85
Beta-xylosidase	*pslG*	Pchs_06212	647493636	2.03
Glycosyltransferase	*pslH*	Pchs_06213	647493640	2.15
Glycosyltransferase	*pslI*	Pchs_06214	647493643	2.55
Acetyltransferase (isoleucine patch superfamily)		Pchs_06216	647493651	2.6
serine hydroxymethyltransferase (EC 2.1.2.1)	*glyA_*	Pchs_06270	496341782	0.53

	1			
Putative GTPases (G3E family)	*yjiA*	Pchs_06274	647493771	1.57
Catalase	*katB*	Pchs_06282	647493790	0.57
ATP-dependent helicase HrpB	*hrpB*	Pchs_06334	647493935	0.61
3-methyladenine DNA glycosylase		Pchs_06375	647494074	0.64
SnoaL-like domain		Pchs_06396	647494150	0.63
Catalase	*katA*	Pchs_06450	647494300	0.45
translation elongation factor 1A (EF-1A/EF-Tu)	*tuf*	Pchs_06497	647485244	0.62
Phospholipase/lecithinase/hemolysin		Pchs_06505	647483767	2.90
Short-chain dehydrogenases of various substrate specificities		Pchs_06514	647483795	0.62
N-acetyl-gamma-glutamyl-phosphate reductase (EC 1.2.1.38)	*argC*	Pchs_06518	647483803	0.61
hypothetical protein [Pseudomonas chlororaphis]			489267827	0.54

Supplementary Table S3. Primers used in this study

Primer names	Primer sequences (5'-3')	Applications
**Primers for RT-qPCR**		
RpoD-F	TGGCAATGAAGTGGACGAAA	*rpoD* gene
RpoD-R	TTCTGCTGGCAACGGATGAT	
PhzA-F	GTACATGCGCACTAACGGCA	*phzA* gene
PhzA-R	AGCATTTCTCCAGCCATTCG	
PhzE-F	CCGACGAGCTGTACATGGTG	*phzE* gene
PhzE-R	GCGCCATTTCCTTGAGGTAA	
PhzD-F	CGACCTGCCCATCAACCTC	*phzD* gene
PhzD-R	CCACTTGTTCACGCAGGG	
PstS-F	CTTCGGTGGTCCAGTCCATC	*pstS* gene
PstS-R	GATGAAGTCGCTGCTGCCTT	
Pchs_00644-F	GTGGTGAAGAACCAGCAGGC	Pchs_00644 gene
Pchs_00644-R	GTGAACTGCTCATAGCGCCC	
PvdJ-F	GGCCTGAGCGACAACTTCTT	*pvdJ* gene
PvdJ-R	GCAGACGCACGAAGTCTTCC	
NrtA-F	TGGTTCATGACCCAGTTCCG	*nrtA* gene
NrtA-R	GGCGGCTTCACGATAGAGCT	
NtrC-F	CTGTTCAACATGTGGTCGCC	*ntrC* gene
NtrC-R	TACATCTCCCAGCCCGAGTC	
GlnA-F	TCGAAGCCTCCGACATGATC	*glnA* gene
GlnA-R	AAGGCTCGATGATGTCGCAC	
ImpC-F	CCTCAACGTCTCCAAGCAGG	*impC* gene
ImpC-R	GGCCGTATTCAGCGGTGTAG	
Hcp-F	AATCCTCCCCCAACCTGATG	*hcp* gene
Hcp-R	TCAGGTACTCCACCGGATCG	
**Primers for gene deletion**		
HS1F1	AAGAATTCATGATGGTGCCGTCCTTCTTC	T6SS-HS1 deletion
HS1R1	GTTGTTCATCCACGCCGAGAA	
HS1F2	TCGGCGTGGATGAACAACACGCCTCCATCAGCCAGTT	
HS1R2	GCTCTAGACTATGGTCAGCGGGAAGTAGC	
HS2F1	AAGAATTCAACTACCGTCTCAAGTGCCATC	T6SS-HS2 deletion
HS2R1	GCCTCGTATTCGCTGGAGAAG	
HS2F2	CTCCAGCGAATACGAGGCGCCGAAGAACACCGCATCA	
HS2R2	GCTCTAGATCACCGTTTCCACCGAGTAGT	
HS3F1	AAGAATTCTACTGCTTGCCGTGGTGCT	T6SS-HS3 deletion
HS3R1	TCGCCAAGGACGTGCTCAA	
HS3F2	TGAGCACGTCCTTGGCGACTACAGCAGTTCCGTGACAAGT	
HS3R2	GCTCTAGACCTACCGTCCTCCGTCCTTT	

Supplementary Figures and Legends

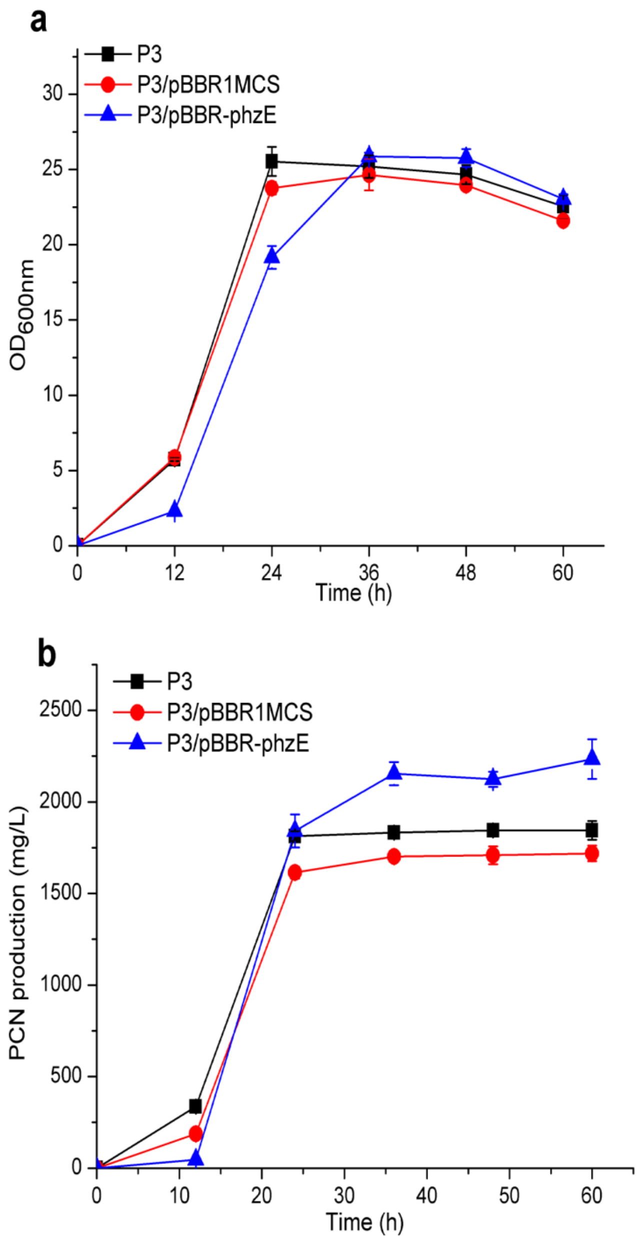

Supplementary Figure S1. Overexpression of *phzE* gene in mutant P3. The growth (a) and phenazine-1-carboxamide production (b).

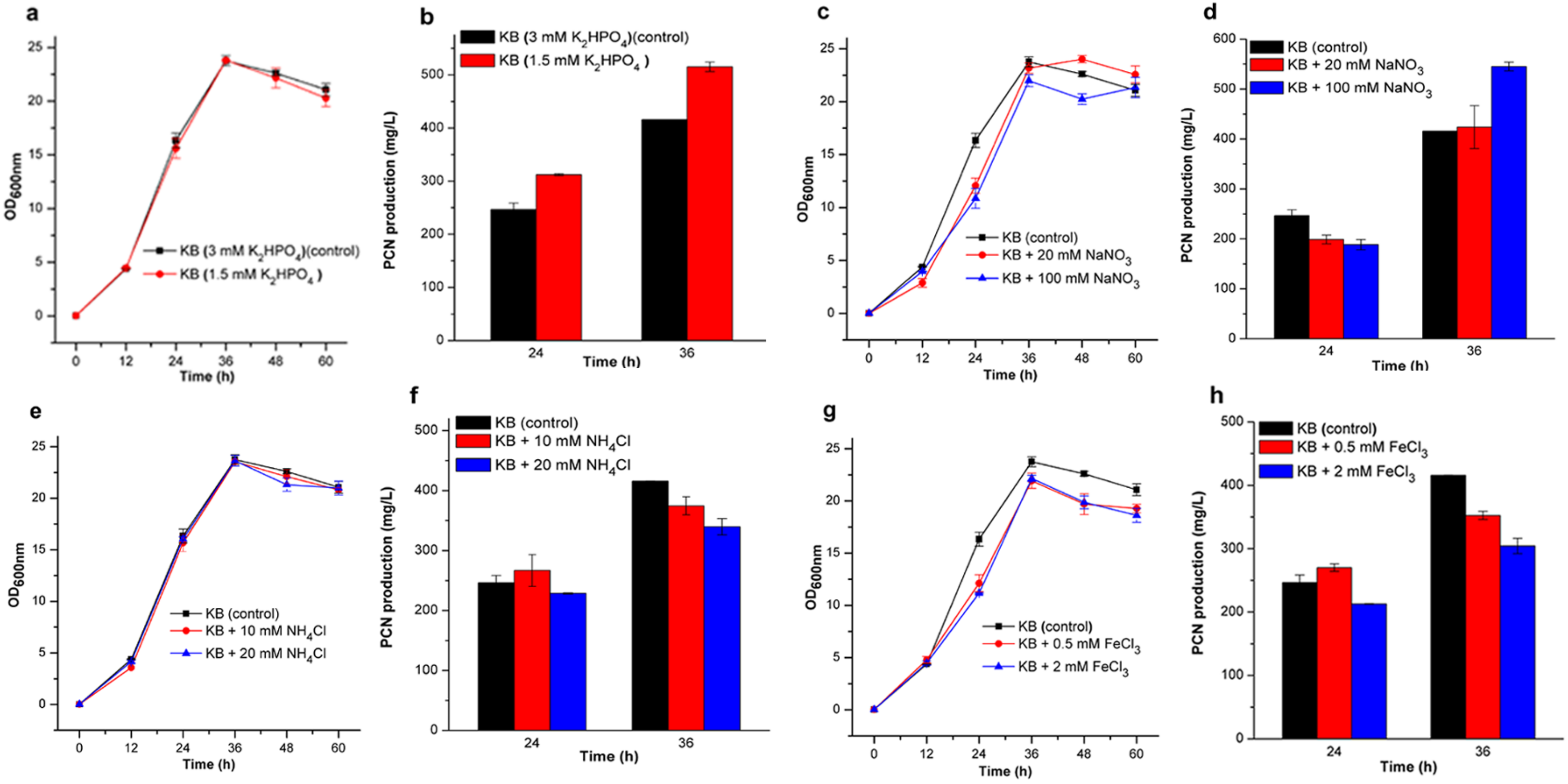

Supplementary Figure S2. Influence of inorganic ions on the growth and phenazine-1-carboxamide (PCN) production. The growth and

PCN production of wild-type strain HT66 in KB medium amended with different concentrations of K_2HPO_4 (a) and (b), $NaNO_3$ (c) and (d), NH_4Cl (e) and (f); $FeCl_3$ (g) and (h), respectively. Experiments were performed at least three times with similar results. Data of one of the experiments are shown.
